# Inflammatory cytokine-primed MSC-derived extracellular vesicles ameliorate acute lung injury via enhanced immunomodulation and alveolar repair

**DOI:** 10.1186/s13287-025-04576-z

**Published:** 2025-08-22

**Authors:** Jongwon Jeong, Jun-Kook Park, Jiwon Shin, Inseong Jung, Hyun-Woo Kim, Anyeseu Park, Hanchae Cho, Sung-Min Kang, Sanghee Shin, Eunju Park, Jisuk Kim, Soojeong Noh, Yongdeok Ahn, Do-Kyun Kim, Jeong Yoon Lee, Daeha Seo, Moon-Chang Baek, Kyungmoo Yea

**Affiliations:** 1 Department of New Biology, Daegu Gyeongbuk Institute of Science and Technology (DGIST), Daegu, 42988 Republic of Korea; 2 Korea Zoonosis Research Institute, Jeonbuk National University, Iksan, 54531 South Korea; 3 The Laboratory of Viromics and Evolution, Zoonosis Research Institute, Jeonbuk National University, 820-120 Hana-ro, Iksan-si, 54531 Jeollabuk-do Republic of Korea; 4 Department of Biomedical Science, School of Medicine, Kyungpook National University, Daegu, 41944 Republic of Korea; 5 Department of Molecular Medicine, Cell and Matrix Research Institute (CMRI), School of Medicine, Kyungpook National University, Daegu, 41944 Republic of Korea; 6 Department of Physics and Chemistry, Daegu Gyeongbuk Institute of Science and Technology (DGIST), Daegu, 42988 Republic of Korea; 7 New Biology Research Center, Daegu Gyeongbuk Institute of Science and Technology (DGIST), Daegu, 42988 Republic of Korea

**Keywords:** Acute lung injury (ALI), Mesenchymal stem cells (MSCs), Extracellular vesicles (EVs), Priming

## Abstract

**Background:**

Acute lung injury (ALI) is characterized by excessive inflammation and alveolar damage, arising from pathogens or systemic insults such as sepsis, and can progress to severe acute respiratory distress syndrome (ARDS). Despite its severity, effective pharmacological treatments remain unavailable, and current clinical interventions are limited to supportive care such as mechanical ventilation. Mesenchymal stem cell-derived extracellular vesicles (MSC-EVs) have emerged as promising candidates for lung repair, but insufficient immunosuppressive capacity often limits their efficacy.

**Methods:**

Human adipose-derived mesenchymal stem cells (hADMSCs) were primed with IFN-γ and TNF-α to enhance the immunomodulatory properties of their secreted EVs. We characterized unprimed control MSC-EVs (C-MEVs) and primed MSC-EVs (P-MEVs) by transmission electron microscopy, nanoparticle tracking analysis, and western blotting for EV markers. Functional assays in THP-1 and A549 cells examined anti-inflammatory potency and barrier regeneration against lipopolysaccharide (LPS)-induced damage. A preclinical mouse model of LPS-induced ALI was used to evaluate inflammatory cytokine expression, immune cell infiltration, pulmonary edema, and vascular leakage. Finally, severe acute respiratory syndrome coronavirus 2 (SARS-CoV-2)-infected Vero E6 cells were tested whether P-MEVs could mitigate the inflammatory damage characteristic of virus-triggered acute lung injury.

**Results:**

Primed hADMSCs exhibited elevated expression of immunosuppressive molecules (e.g., COX-2, IDO, TSG-6), without changing EV morphology or yield. P-MEVs mitigated LPS-induced inflammation more effectively than C-MEVs in THP-1 and A549 cells. In vivo, P-MEVs more robustly attenuated inflammatory cytokines, immune cell recruitment, and lung injury markers in mice challenged with LPS. In SARS-CoV-2-infected Vero E6 cells, P-MEVs suppressed cytopathic effects and inflammatory responses more potently than C-MEVs. Mechanistic analyses revealed that these enhancements were associated with elevated miRNA levels, including miR-221-3p, involved in inhibiting inflammatory pathways.

**Conclusion:**

Inflammatory cytokine priming substantially augments the immunomodulatory and tissue-regenerative efficacy of hADMSC-derived EVs, offering superior therapeutic effects in ALI models and promising activity against SARS-CoV-2-induced lung damage. These findings underscore the therapeutic potential of P-MEVs as an innovative, cell-free platform for treating severe pulmonary disorders, including ARDS.

**Supplementary Information:**

The online version contains supplementary material available at 10.1186/s13287-025-04576-z.

## Background

Acute lung injury (ALI) is characterized by an excessive inflammatory response and alveolar damage, and can arise from various causes, including bacterial or viral infections, sepsis, and trauma [1]. The global crisis of the COVID-19 pandemic has starkly highlighted the devastating impact of virus-induced ALI, which frequently progresses to acute respiratory distress syndrome (ARDS), a condition that typically requires intensive care unit admission due to its severity [2–5]. In ALI, pathogens or endotoxins infiltrate the alveolar space, activating immune and epithelial cells and triggering a cytokine storm [6]. This heightened inflammatory cascade increases vascular permeability, resulting in pulmonary edema and severe respiratory failure [1, 4–6]. Currently, there are no efficacious pharmacological therapies for ALI, and no definitive treatment exists for severe ARDS beyond supportive measures, including mechanical ventilation [6–10]. Consequently, there is an urgent need to develop effective therapeutic interventions that suppress the cytokine storm and foster lung repair mechanisms for managing ALI.

Mesenchymal stem cell-derived extracellular vesicles (MSC-EVs) have emerged as a promising cell-free therapeutic option for ALI due to their ability to modulate immune responses and facilitate tissue regeneration [11, 12]. MSC-EVs, which are enriched with miRNAs, proteins, and growth factors, have shown efficacy in preclinical studies by alleviating cytokine storm-related complications such as alveolar inflammation, edema, and epithelial injury [13, 14]. In addition, MSC-EVs offer several advantages over cell-based therapies, including reduced immunogenicity, ease of storage, and the avoidance of risks associated with MSC transplantation—such as pulmonary embolism [15, 16]. Despite these favorable therapeutic properties, MSC-EVs face clinical limitations due to their insufficient immunomodulatory capacity, resulting in suboptimal efficacy [17–19]. This limitation highlights the need for strategies that enhance the immunomodulatory functions of MSC-EVs to advance their clinical translation.

Recent studies suggest that extracellular vesicles (EVs) derived from mesenchymal stem cells (MSCs) preconditioned with inflammatory cytokines such as IFN-γ and TNF-α exhibit enhanced anti-inflammatory and immunosuppressive properties compared to EVs from unprimed MSCs. The immunomodulatory functions of MSCs are highly adaptable and responsive to the microenvironment, specifically influenced by the particular environment of the host cells [20]. Under inflammatory conditions, MSCs upregulate the secretion of key anti-inflammatory mediators, including COX-2, IDO, and TSG-6, which are critical in suppressing excessive immune responses [21, 22]. Therefore, MSCs can be primed to enhance their immunosuppressive functions by replicating these inflammatory microenvironments in vitro, thereby significantly improving the therapeutic properties of the released EVs [23, 24]. Thus, inflammatory priming represents a valuable strategy to overcome the current limitations of MSC-EVs in ALI treatment.

In this study, we investigated the enhanced immunosuppressive and tissue-regenerative properties of EVs released from human adipose-derived mesenchymal stem cells (hADMSCs) stimulated with IFN-γ and TNF-α. We then evaluated the anti-inflammatory and tissue-regenerative effects of these primed MSC-EVs (P-MEVs) in a preclinical mouse model of ALI induced by lipopolysaccharide (LPS). To investigate the broader applicability of P-MEVs, we also extended our investigation as an exploratory study to an in vitro SARS-CoV-2 infection model, assessing their potential against lung cell injury of viral etiology. Finally, mechanistic analysis revealed that P-MEVs exhibit elevated expression of miRNAs that negatively regulate inflammatory pathways, which play a pivotal role in their enhanced immunomodulatory function.

## Materials and methods

### Cell culture

hADMSCs were purchased from CEFObio (Seoul, Republic of Korea). THP-1 (TIB-202), A549-Luc2 (CCL-185-LUC2), and Vero E6 (CRL-1586) were obtained from the American Type Culture Collection (Manassas, VA, USA). hADMSCs were cultured in CEFOgro human MSC growth medium containing 10% supplements and 0.5% penicillin and streptomycin (CEFOgro-MSC, CEFObio) and used between passages 2 and 5. THP-1 and A549-Luc2 were cultured in Roswell Park Memorial Institute medium (Hyclone, Logan, UT, USA) containing 10% heat-inactivated fetal bovine serum (FBS, Gibco; Thermo Fisher Scientific, Waltham, MA, USA) and 1x Antibiotic-Antimycotic solution (AA, CA002-010, GenDEPOT, Katy, TX, USA). Vero E6 cells were cultured in Dulbecco’s modified Eagle’s medium (DMEM) supplemented with 10% heat-inactivated FBS and 1x AA. All cells were maintained at 37 °C in a humidified incubator with 5% CO_2_.

### Inflammatory priming of hADMSCs with IFN-γ and TNF-α

To stimulate the hADMSCs with inflammatory factors, cells were seeded in 100 mm cell culture dishes (1 × 10^5^ cells/dish), and after 24 h, the supernatant was replaced with fresh culture medium containing IFN-γ (10 ng/mL, 300-02-100UG; Peprotech, Rocky Hill, NJ, USA) and TNF-α (15 ng/mL, 300–01 A-50UG; Peprotech) as recommended by the International Society of Cell and Gene Therapy (ISCT) [25]. hADMSCs that were not primed using cytokines served as controls. After 24 h, the hADMSC morphologies were examined by light microscopy, and their viability was measured by mitochondrial activity using the CellTiter 96 AQueous One Solution Cell Proliferation Assay System (MTS assay, G3582; Promega, Madison, WI, USA). Further, hADMSC counting was performed using an automated cell counter (LUNA-II; Logos Biosystems, Gyeonggi-do, Republic of Korea). In addition, cells were harvested for RNA and protein extraction.

### Isolation of hADMSC-derived EVs

The EVs were isolated from both control and primed hADMSCs. Control and primed hADMSCs (1 × 10^5^ cells/100 mm dish) were maintained in 10 mL of MSC growth medium without supplements for 48 h. Cell culture media were collected twice every 24 h. The collected conditioned media were serially centrifuged at 300 x g for 5 min, 2500 x g for 20 min, and 10,000 x g for 30 min to remove cell debris and large vesicles, and the supernatants were then filtered through 0.2 μm syringe filters. To remove soluble proteins and antibiotics, the resulting supernatants were subjected to a tangential flow filtration (TFF) system [26] equipped with 0.05 μm PS hollow fiber (D02-S05U-05-S; Repligen, Waltham, MA, USA). The supernatants were continuously circulated through a membrane filter system at a pressure of less than 20 psi and exchanged with two volumes of phosphate-buffered saline (PBS). The EVs were harvested in a final volume of approximately 10 mL. The prepared EVs were stored at − 80 °C until further use. Characterization of hADMSC-derived EVs was performed according to the minimal information for studies of extracellular vesicles (MISEV2023) guidelines [27].

### Nanoparticle tracking analysis (NTA)

Immediately after isolating the MSC-EVs, the size distribution and concentration were measured using NanoSight LM10 (Nanosight, Salisbury, UK). To examine the movement and morphology of the nanoparticles, a monochromatic laser beam was applied at 405 nm, and a 30 s video was acquired at a rate of 30 frames/s and a camera level of 9. Captured vesicle movements were analyzed to measure particle size and concentration using the NTA software (version 2.3, Nanosight). NTA post-acquisition settings were optimized and remained constant between samples.

### Transmission electron microscopy (TEM)

A fixative solution was prepared by combining 2.5% glutaraldehyde (Grade I, 25% in H_2_O; Sigma-Aldrich, St. Louis, MO, USA) and 1.5% formaldehyde (ACS reagent, 37% by weight in H_2_O, containing 10–15% methanol as a stabilizer; Sigma-Aldrich) in 0.2 M sodium cacodylate buffer (pH 7.2, adjusted with HCl). The emulsion from the EVs was mixed with the fixative in a 1:1 v/v ratio. The mixture was incubated at 4 °C for 30 min, with pipetting performed every 10 min to ensure uniform distribution. After fixation, the EV solution was applied to a plasma-cleaned gold (Au) TEM grid (200 mesh; TED Pella, Redding, CA, USA). The grid was maintained at 4 °C for 10 min before the excess solution was removed using filter paper. Next, the TEM grid was washed using 0.2 M sodium cacodylate buffer before deionized water (DIW) was used to remove the residual fixative. For negative staining, 1% uranyl acetate (98–102%; Electron Microscopy Sciences, Hatfield, PA, USA) was applied to the EV-coated grid and left for 3 min before removal. The stained EVs were washed using DIW and air-dried at room temperature. The prepared sample was then visualized using a transmission electron microscope (Hitachi HF-3300, Tokyo, Japan), and the resulting images were analyzed using ImageJ software (NIH, Bethesda, MD, USA).

### Western blot analysis

Total proteins were extracted from the hADMSCs, MSC-EVs, mouse lung tissue, and Vero E6 using cell lysis buffer (9803; Cell Signaling Technology, Danvers, MA, USA) supplemented with a protease/phosphatase inhibitor cocktail (5872; Cell Signaling Technology), on ice. After centrifugation at 13,000 rpm for 10 min, the supernatants were collected, and protein concentrations were quantified using the bicinchoninic acid protein assay kit (23225; Thermo Fisher Scientific). In total, 20 µg of protein was denatured in sodium dodecyl sulfate (SDS) sample buffer containing 2% 2-mercaptoethanol and boiled for 10 min. SDS-PAGE was performed to separate each sample before the proteins were transferred to nitrocellulose membranes (IB301002; Invitrogen; Thermo Fisher Scientific). The membranes were blocked using 5% BSA in 0.1% Tween-20 in Tris-buffered saline for 1 h and incubated with primary antibodies at 4 °C overnight. Then, the blots were probed using the specified HRP-conjugated secondary antibody. The following primary antibodies (1:1000): anti-GM130 antibody (610822; BD Life Sciences, Franklin Lakes, NJ, USA), anti-ALIX antibody (ab117600; Abcam, Cambridge, UK), anti-TSG101 antibody (ab30871; Abcam), anti-β-Actin antibody (4967; Cell Signaling Technology), anti-COX2 antibody (12282; Cell Signaling Technology), anti-Stat3 antibody (12640; Cell Signaling Technology), anti-p-Stat3 antibody (9145; Cell Signaling Technology), anti-NF-κB p65 antibody (8242; Cell Signaling Technology), anti-p-NF-κB p65 antibody (3033; Cell Signaling Technology), and secondary antibodies (1:2000): anti-rabbit IgG, HRP-conjugated antibody (7076; Cell Signaling Technology), anti-mouse IgG, HRP-conjugated antibody (7074; Cell Signaling Technology) were used and the protein bands were detected using chemiluminescent substrate (34095; Thermo Fisher Scientific). The ChemiDoc XRS + system (Bio-rad, Hercules, CA, USA) was used to quantify protein levels, and the relative protein expressions were measured using ImageJ software (NIH).

### Quantitative reverse transcription-polymerase chain reaction (qRT-PCR)

Total RNA was extracted from hADMSCs, THP-1, A549, mouse lung tissue, and Vero E6 using the MiniBEST Universal RNA Extraction kit (9767; TaKaRa, San Jose, CA, USA). Complementary DNA strands were synthesized from the isolated RNA using the PrimeScipt First Strand cDNA Synthesis kit (6110 A; TaKaRa). Gene expression was assessed by qRT-PCR using the TB Green Premix Ex Taq kit (RR420A; TaKaRa). Triplicate reactions were performed in a 20 µL volume with specific primer pairs using the StepOnePlus qRT-PCR System (Applied Biosystems; Thermo Fisher Scientific). The mRNA expression levels were normalized to *glyceraldehyde-3-phosphate dehydrogenase* (*GAPDH*). A comparative threshold cycle (Ct) analysis was used to calculate the relative mRNA amount of samples after normalization with *GAPDH*. Data are expressed as modified 2^(−ΔΔCt)^ values from the original Ct values. The primer sequences used in this analysis are listed in Table S1.

### In vitro studies with THP-1 macrophage-like cells and A549 lung epithelial cells

THP-1 cells were cultured in a medium supplemented with 50 nM phorbol-12-myristate 13-acetate (PMA; P8139; Sigma-Aldrich) to induce differentiation into adherent macrophage-like cells. After 48 h of PMA treatment, THP-1 macrophage-like cells were washed and allowed to grow in PMA-free culture medium for the next 24 h. To investigate the effect of MSC-EVs on LPS-induced inflammatory responses, THP-1 macrophage-like cells and A549 cells were pretreated with different concentrations of MSC-EVs (10, 100, and 1000 EVs/cell) for 2 h to ensure cellular uptake and then activated with LPS from E. coli 0111:B4 (L2630; Sigma-Aldrich). RNA was isolated after 6 h for qRT-PCR, and the culture supernatant was harvested for ELISA after 24 h.

### Enzyme-linked immunosorbent assay (ELISA)

Cell culture supernatants and lung tissue homogenates were collected after centrifugation at 550 x g for 10 min. The TNF-α, IL-1β, and IL-6 levels in THP-1 and A549 supernatants and mouse lung tissue were measured using ELISA kits (88-7326 for human TNF-α, 88-7261 for human IL-1β, 88-7066 for human IL-6, 88-7324 for mouse TNF-α, 88-7013 for mouse IL-1β, 88-7064 for mouse IL-6; Invitrogen; Thermo Fisher Scientific) according to the manufacturer’s recommendations. Absorbance was measured at 450 nm using a VersaMax microplate reader (Molecular Devices, San Jose, CA, USA).

### Cell viability assay

The MTS assay was used to analyze the effect of MSC-EVs on the viability of LPS-treated A549 cells. Here, A549 cells (5 × 10^3^ cells/well) were seeded in a 96-well plate and allowed to adhere for 16 h. Subsequently, to facilitate internalization, the cells were pretreated with different concentrations of MSC-EVs (10, 100, and 1000 EVs/cell) for 2 h. Following pretreatment, the cells were activated using LPS (500 µg/mL) for 24 h.

To investigate the cytotoxicity of MSC-EVs and LPS on THP-1 macrophage-like cells, A549 cells, and Vero E6 cells, each cell type was seeded at a density of 5 $\:\times\:$ 10^4^ cells/mL (100 µL) in a 96-well plate for 16 h. The cells were then incubated with different concentrations of MSC-EVs and LPS for 24 h. Untreated cells were used as control groups. MTS reagent (20 µL) was added to each well and incubated at 37 °C for 2 h, and the absorbance was measured at 490 nm using a VersaMax Microplate Reader (Molecular Devices).

### In vitro epithelial cell permeability assay

A549 cells (1 × 10^5^ cells per well) were cultured on 0.4 μm pore size Transwell inserts (353095; Falcon, Corning, NY, USA) in a 24-well plate until a confluent monolayer was formed. The cells were then treated with different concentrations of MSC-EVs (10, 100, and 1000 EVs/cell) for 2 h to facilitate internalization. Next, LPS (500 µg/mL) was added to each well for an additional 24 h, after which the upper chamber medium was replaced with a medium containing 500 µg/mL FITC-conjugated 40 kDa dextran (53379; Sigma-Aldrich). After 3 h, the fluorescence intensity in the lower chambers was measured using an EnVision multimode plate reader (PerkinElmer, Waltham, MA, USA) at excitation and emission wavelengths of 485 nm and 530 nm, respectively.

### Animals and LPS-induced ALI mouse model

All in vivo experimental protocols were ethically approved by the DGIST Institutional Animal Care and Use Committee (IACUC; approval number: DGIST-IACUC-24010205-0000). The work has been reported in line with the ARRIVE guidelines 2.0. Male C57BL/6 mice, 8 weeks of age, were purchased from Orient Bio (Seongnam-si, Republic of Korea) and maintained under standard conditions (20–24 °C, 45–65% relative humidity) with a 12 h light–dark cycle. For intravenous injections of LPS or EVs, mice were briefly anesthetized with 2% isoflurane via inhalation. Euthanasia was performed by CO_2_ inhalation in accordance with institutional guidelines. To establish the LPS-induced ALI mouse model, randomized mice received an intravenous injection of 5 mg/kg LPS, while control mice received an equal volume of PBS. LPS-treated mice received a daily dose of 150 µL MSC-EVs (6.0 $\:\times\:$ 10^9^ particles) by tail vein injection (6.0 $\:\times\:$ 10^9^ particles) at 4 h, 24 h, and 48 h after LPS treatment. Control and LPS-only treated groups received 150 µL of PBS at the same time points. (10 mice/group, total 40 mice per experiment). Mice had *ad libitum* access to a normal chow diet and water, and body weight was monitored daily. Mice were euthanized 72 h after LPS treatment, and lung tissues were collected for subsequent experiments. One portion of lung tissue was removed from five mice in each group and fixed with 4% paraformaldehyde (PFA) for histological analysis. Another portion was frozen in liquid nitrogen for RNA and protein extraction, and a third portion of lung tissue was used for cell isolation. For the other five mice in each group, the left lung was used to measure the wet-to-dry (W/D) ratios, and the right lung was used for Evans blue extravasation analysis. Mice that died were excluded from the statistical analysis.

### Flow cytometry

Lung tissue was minced into small pieces of less than 1 mm^3^ on ice, placed on a strainer in a 50 mL conical tube, and crushed with the blunt end of a 10 mL syringe. Samples were washed with RPMI and resuspended in 500 µL of Red Blood Cell Lysing Buffer Hybri-Max (R7757; Sigma-Aldrich) for 5 min. Then, 4.5 mL of PBS was added for washing, and the samples were centrifuged at 550 × g to obtain the cells. All cells were fixed using 4% PFA mixed in PBS and incubated at room temperature for 10 min. After blocking, the cells were stained with the following fluorescence-conjugated antibodies: anti-CD45-APC-Cy7 antibody (103115, clone 30-F11; Biolegend, San Diego, CA, USA), anti-Ly6G-FITC antibody (127605, clone 1A8; Biolegend), anti-Siglec-F-BV421 antibody (565934, clone E50-2440; BD Life Sciences), anti-F4/80-eFluor™ 506 antibody (69-4801-80, clone BM8; Invitrogen; Thermo Fisher Scientific), anti-CD11b-PE antibody (24965; Cell Signaling Technology), and anti-CD11c-APC antibody (33293; Cell Signaling Technology). Cell populations were analyzed using CytoFLEX (Beckman Coulter, Brea, CA, USA) and the data were analyzed using CytExpert software (Beckman Coulter).

### Lung W/D ratio

The severity of pulmonary edema was assessed by determining the W/D ratio in the lung. After the lungs were removed and washed using PBS, surface moisture was removed with clean filter paper, and the wet weight was recorded. The tissue was then placed in an oven at 60 °C for 48 h to obtain the dry weight. The W/D ratio was calculated to evaluate the moisture content of the lung tissue.

### Evans blue dye extravasation

To assess lung barrier permeability, mice were injected intravenously 2 h before euthanasia with 100 µL of 1% Evans Blue (E2129; Sigma-Aldrich) mixed in PBS. The excised lung tissues were weighed and incubated with formamide at 60 °C for 48 h. After incubation, the samples were centrifuged at 12,000 x g for 20 min. The optical density of the supernatant was measured at 620 nm using a VersaMax Microplate Reader (Molecular Devices). The extravasated Evans blue dye concentration was determined using a standard curve and expressed as µg of Evans blue dye per g of lung tissue.

### Hematoxylin and Eosin (H&E) staining

Harvested lungs were fixed in 4% (v/v) paraformaldehyde overnight, dehydrated, cleared, and embedded in paraffin. Paraffin-embedded tissues were then sectioned into 5 μm slices. Lung tissue sections were deparaffinized, rehydrated, and then stained with H&E. After staining and dehydration, the glass slides containing the tissue sections were mounted in mounting solution and photographed under a Nikon Eclipse Ni-E light microscope (magnification, x40 and x200; Nikon Corp., Tokyo, Japan).

### Virus amplification

The SARS-CoV-2 isolate (NCCP No. 43326) was obtained from the National Culture Collection for Pathogens (Osong, Republic of Korea). To amplify a virus stock, the Vero E6 cells (2 × 10^6^ cells in T75 culture flasks) were infected with the virus at a multiplicity of infection (MOI) of 0.1. The virus supernatant was harvested with a 50% cytopathic effect (CPE). The plaque assay was employed to perform the virus titration in Vero E6 cells (6-well plate, 4 × 10^5^ cells/well). All experiments using the SARS-CoV-2 strain were conducted in the Biosafety Laboratory Level 3 facility at the Korea Zoonosis Research Institute (KoZRI), Jeonbuk National University (Iksan, Republic of Korea).

### SARS-CoV-2 infection in Vero E6 cells

Vero E6 cells (1.5 × 10^5^ cells per well) were seeded in 6-well plates overnight. To avoid overinfection and excessive cell toxicity, an MOI of 0.003 was used and the cells were inoculated with 200 µL of SARS-CoV-2. The non-infected control received DMEM media only. After incubation at 37 °C with 5% CO_2_ for 1 h, rocking every 15 min, the inoculum was removed. The cells were washed once with DMEM media and replenished in 2 mL of DMEM containing 2% FBS, 1x AA, and various concentrations of MSC-EVs (10, 100, and 1000 EVs/cell) for an additional 48 h. Images confirming CPE were captured using a microscope (DMi8; Leica, Wetzlar, Germany). Cell pellets were obtained by removing the supernatant, washing with 1 mL of cold PBS, and scraping the monolayer with 1 mL of fresh PBS. After centrifugation at 300 x g and 4 °C for 5 min, the cells were frozen at -80 °C for subsequent RNA and protein extraction.

### Small RNA isolation from EVs

RNA was isolated from EVs using TRIzol LS reagent (10296028; Invitrogen; Thermo Fisher Scientific) according to the manufacturer’s instructions. RNA quality was evaluated using an Agilent 2100 bioanalyzer equipped with an RNA 6000 Nano Chip (Agilent Technologies, Santa Clara, CA, USA). RNA quantification was performed using a NanoDrop 2000 spectrophotometer (Thermo Fisher Scientific).

### MiRNA library preparation and sequencing

A small RNA library was prepared using the NEBNext Multiplex Small RNA Library Prep kit (New England Biolabs, Ipswich, MA, USA) according to the manufacturer’s protocol. To construct the library, cDNA synthesis was initiated by adaptor ligation using 1 µg of total RNA from each sample, followed by reverse transcription using adaptor-specific primers. The library was amplified by PCR and purified using the QIAquick PCR Purification kit (Qiagen, Hilden, Germany) and AMPure XP beads (Beckman Coulter). Then, the yield and size distribution of the small RNA libraries were analyzed using an Agilent 2100 Bioanalyzer alongside a high-sensitivity DNA assay (Agilent Technologies, USA). High-throughput sequencing was performed by single-ended 75 sequencing using the NextSeq500 system (Illumina, San Diego, CA, USA).

### MiRNA data analysis

The data were analyzed using the Bowtie2 software tool to generate a bam file (alignment file). Mapping was based on the mature miRNA sequence. Read counts mapped to the mature miRNA sequences were extracted from the alignment file using the bed tool (version 2.25.0) and Bioconductor, which uses the R (version 3.2.2) statistical programming language (R Development Core Team, 2011). These read counts were used to assess the miRNA expression levels, with comparisons between samples performed using the quantile normalization method. miRWalk 2.0 was used for miRNA target exploration. Data mining and visualization were performed using ExDEGA (Ebiogen Inc., Republic of Korea). GO (Gene Ontology) enrichment and KEGG (Kyoto Encyclopedia of Genes and Genomes) pathway analyses were performed for target genes using the online computational resource DIANA-miRPath v4.0 with target selection from TargetScan v.8.0 [28].

### Statistical analysis

All values are presented as the mean ± standard error of the mean (SEM), as indicated in the figure legends. Statistical comparisons between the two groups were performed using an unpaired, two-tailed Student’s *t*-test. Differences between multiple groups were statistically analyzed using a one-way analysis of variance (ANOVA) or two-way ANOVA, with the Holm–Sidak multiple comparison test for post hoc analysis. Values of *p < 0.05, **p < 0.01, ***p < 0.001, and ****p < 0.0001 were applied to indicate statistical significance, and the *p*-values are further specified in the figure legends. Statistical analysis was performed using GraphPad Prism 8 (GraphPad Software, San Diego, CA, USA).

## Results

### Characterization of hADMSC-derived EVs

**Fig. 1:**
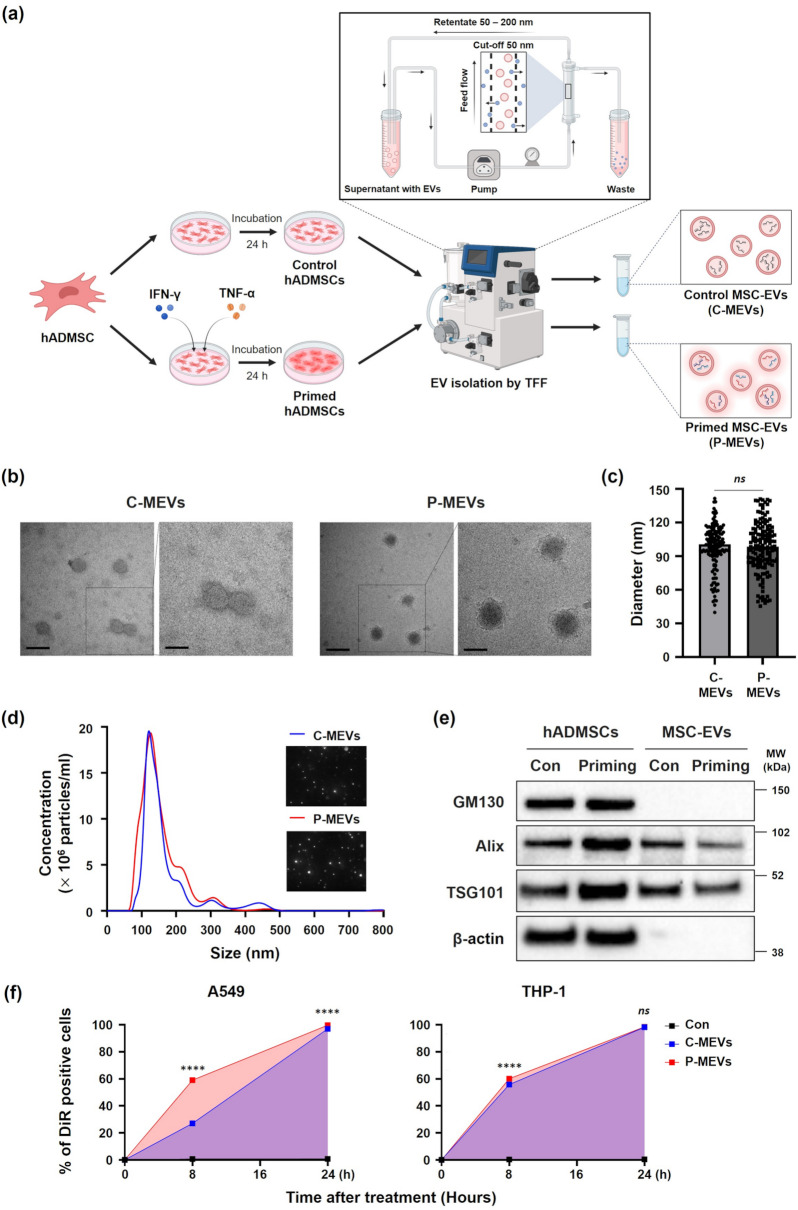
Characterization of hADMSC-derived EVs (**a**) Schematic diagram showing the steps involved in the production of EVs isolated from control (C-MEVs) or primed hADMSCs (P-MEVs). Created using BioRender.com. (**b**) Representative TEM image of EVs derived from control and primed hADMSCs. Black scale bars represent 200 nm (left) and 100 nm (right). (**c**) The average diameter of EVs measured from the images in (**b**). *n =* 150. (**d**) NTA depicting the size distribution and representative video images of C-MEVs and P-MEVs. The observed size range of EVs was 50–200 nm. (**e**) Immunoblotting for representative cell and EV markers in cell lysate and EVs isolated from the indicated samples (Full-length blots are presented in Supplementary Figure S9a). (**f**) Time-course analysis of DiR-labeled EV uptake in A549 and THP-1 cells. Cells were incubated with 1000 EVs/cell, and intracellular fluorescence was quantified by flow cytometry at 0, 8, 16, and 24 h. *n =* 4. Data are presented as the mean ± standard error of the mean (SEM), analyzed by unpaired two-tailed Student’s t-test for (**c**), and by two-way ANOVA followed by the Holm–Sidak multiple comparison test for (**f**). Statistical differences in post hoc tests are indicated as *ns* = not significant, and ****p < 0.0001

We treated hADMSCs with the inflammatory cytokines IFN-γ and TNF-α to enhance the potency of their EVs, following ISCT recommendations [25]. After stimulation, there were no significant differences in cell morphology, proliferation, or viability between control and primed hADMSCs (Figure S1a–c). Nonetheless, the primed hADMSCs displayed significantly increased mRNA levels of COX-2, A20, and TSG-6 (Figure S1d), along with higher COX-2 and IDO protein expression (Figure S1e), indicating enhanced immunosuppressive capacity without altering basic cellular properties. To determine whether inflammatory priming could affect the characteristics of EVs, we first isolated EVs from the culture supernatant of both control and primed hADMSCs using the TFF system (Fig. 1a). TEM revealed that both control MSC-EVs (C-MEVs) and P-MEVs were round and cup-shaped (Fig. 1b) with an average diameter of approximately 95 nm (Fig. 1c). NTA revealed that both C-MEVs and P-MEVs had a similar size distribution of vesicles with no differences in the levels of EV secretion (Fig. 1d). In addition, C-MEVs and P-MEVs were found to have comparable expressions of EV markers, including Alix and TSG101 (Fig. 1e), while no signal was observed for GM130 (Golgi marker), suggesting that the EVs were successfully isolated with minimal contamination. These results indicate that inflammatory priming did not affect the morphology, size, and EV marker expression of hADMSC-derived EVs.

To assess whether inflammatory priming affects the cellular uptake efficiency of EVs, we performed a time-course uptake assay using DiR-labeled C-MEVs and P-MEVs in THP-1 and A549 cells. Flow cytometric analysis revealed that both cell types efficiently internalized EVs, with P-MEVs exhibiting consistently higher fluorescence signals than C-MEVs, particularly at the 8 h time point (Fig. 1e. These results suggest that inflammatory priming enhances the uptake of EVs by recipient cells, which may contribute to their increased therapeutic efficacy.

### P-MEVs ameliorate LPS-induced inflammation and damage in macrophages and lung epithelial cells

**Fig. 2:**
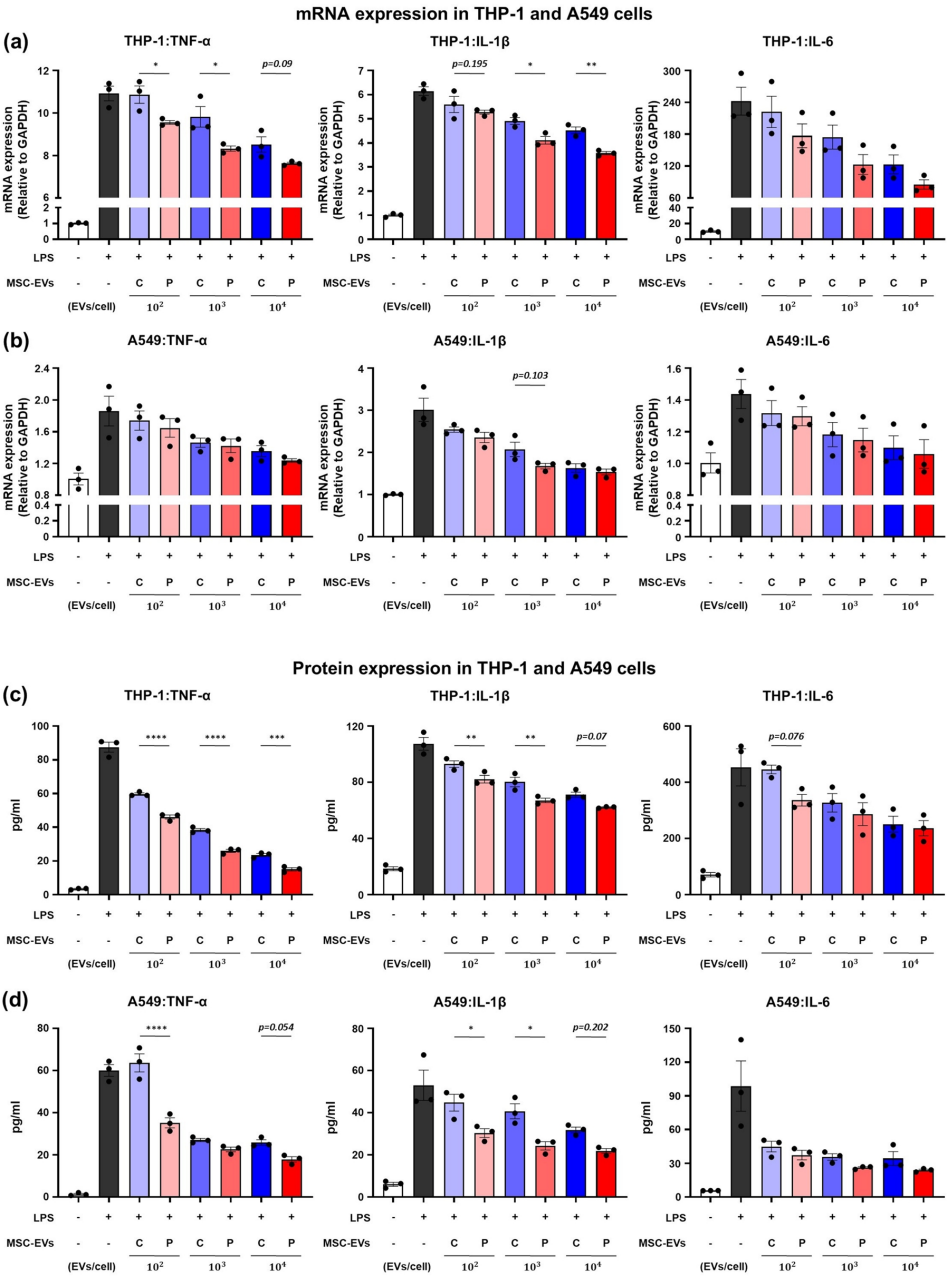
Evaluation of the anti-inflammatory effects of P-MEVs against LPS-induced inflammation in macrophages and lung epithelial cells Macrophages and lung epithelial cells were pretreated with MSC-EVs (C-MEVs or P-MEVs) or PBS for 2 h, followed by exposure to the indicated concentration of LPS for the indicated periods. (**a**) qRT-PCR analysis of relative mRNA expression levels of *TNF-α*, *IL-1β*, and *IL-6* in THP-1 cells 6 h post LPS exposure (5 µg/mL). *n =* 3. (**b**) qRT-PCR analysis of relative mRNA expression levels of *TNF-α*, *IL-1β*, and *IL-6* in A549 cells 6 h post LPS exposure (50 µg/mL). *n =* 3. (**c**) ELISA analysis of TNF-α, IL-1β, and IL-6 levels in THP-1 cells 24 h after LPS exposure (5 µg/mL). *n =* 3. (**d**) ELISA analysis of TNF-α, IL-1β, and IL-6 levels in A549 cells 24 h after LPS exposure (50 µg/mL). *n =* 3. C, control; P, primed. Data are presented as the mean ± SEM, analyzed by one-way ANOVA, followed by the Holm–Sidak multiple comparison test. Statistical differences in post hoc tests are indicated as *p < 0.05, **p < 0.01, ***p < 0.001, and ****p < 0.0001

ALI is a prevalent pulmonary condition marked by epithelial and endothelial barrier disruption, and neutrophilic infiltration [1, 6]. Macrophages play a key role in mediating inflammatory responses by activating proinflammatory pathways [29]. In particular, LPS is widely recognized as a potent proinflammatory stimulant for macrophages and lung epithelial cells [30]. It is also well known that proinflammatory cytokines such as TNF-α, IL-1β, and IL-6 are key mediators in LPS-induced inflammatory responses [31]. Based on preliminary experiments (Figure S2a, b), we treated THP-1 cells with 5 µg/mL LPS and A549 cells with 50 µg/mL LPS to induce robust inflammation without excessive cytotoxicity. Neither C-MEVs nor P-MEVs impaired cell viability at any dose (Figure S2c, d). Under these conditions, LPS significantly elevated TNF-α, IL-1β, and IL-6 mRNA levels in both THP-1 and A549 cells (Fig. 2a, b), providing a suitable platform to assess the immunomodulatory capacity of MSC-EVs. Co-treatment with C-MEVs or P-MEVs attenuated LPS-induced inflammation in a dose-dependent manner, with P-MEVs displaying a more pronounced effect than C-MEVs (Fig. 2a, b). To further evaluate secreted cytokines, conditioned media were analyzed by ELISA. Consistent with the transcriptional data, LPS stimulation significantly increased TNF-α, IL-1β, and IL-6 levels. In both LPS-induced THP-1 and A549, treatment with C-MEVs or P-MEVs suppressed these proinflammatory mediators in a dose-dependent manner, with P-MEVs again showing greater efficacy than C-MEVs (Fig. 2c, d).

**Fig. 3:**
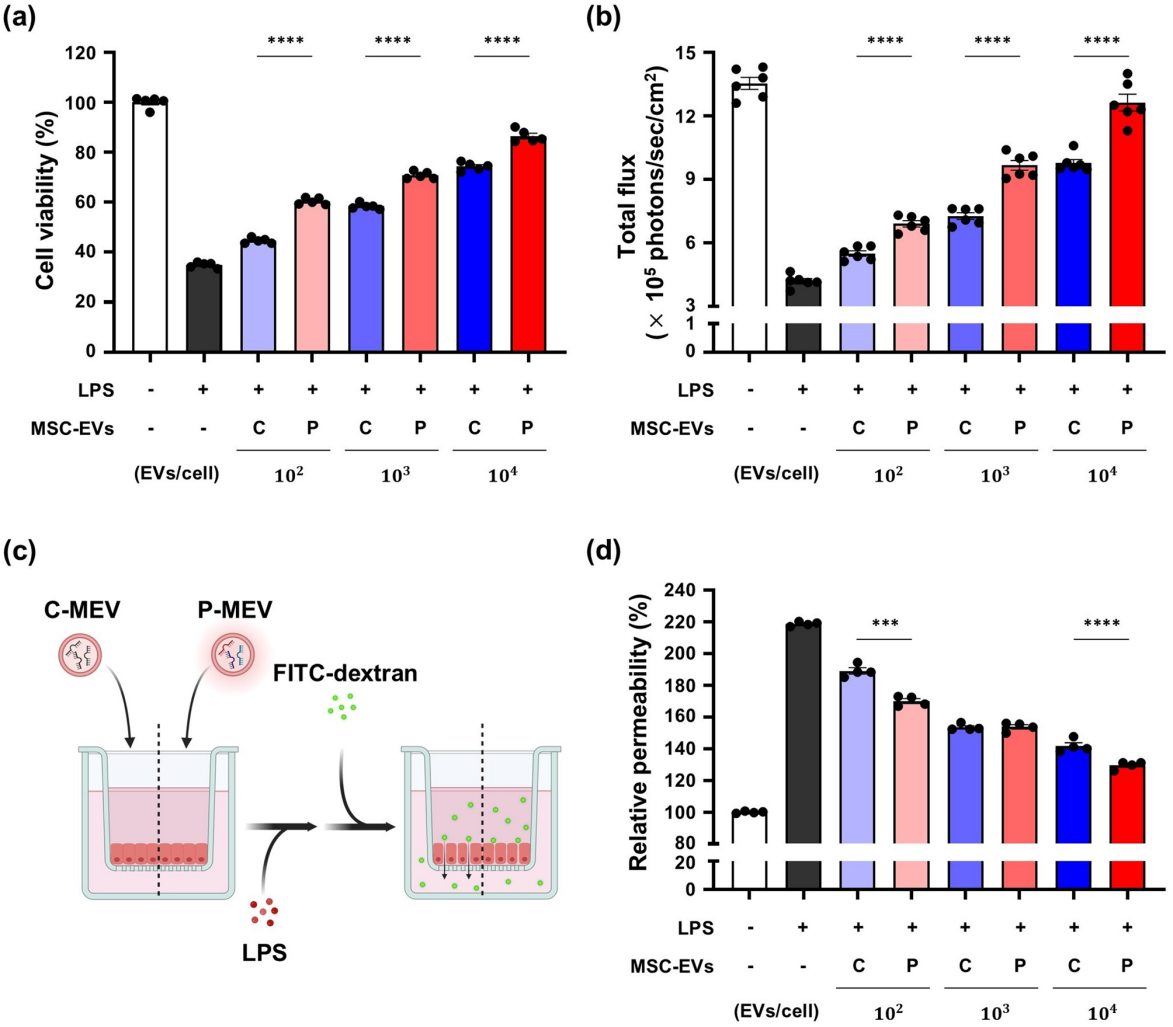
Evaluation of the tissue regenerative effects of P-MEVs against LPS-induced damage in lung epithelial cells Lung epithelial cells were pretreated with MSC-EVs (C-MEVs or P-MEVs) or PBS for 2 h, followed by exposure to LPS (500 µg/mL) for 24 h. (a, b) Assessment of cell viability in A549 cells by (**a**) MTS assay (*n =* 5) and (**b**) in vitro bioluminescence assay (*n =* 6). (**c**, **d**) Assessment of epithelial permeability in A549 cells by measuring the fluorescence intensity of FITC–dextran. (**c**) Experimental scheme (created using BioRender.com) and (**d**) relative permeability (*n =* 4). C, control; P, primed; C-MEV, control MSC-EV; P-MEV, primed MSC-EV. Data are presented as the mean ± SEM, analyzed by one-way ANOVA, followed by the Holm–Sidak multiple comparison test. Statistical differences in post hoc tests are indicated as ***p < 0.001 and ****p < 0.0001

Next, we evaluated the ability of MSC-EVs to restore lung epithelial barrier integrity by measuring cell viability and epithelial permeability in LPS-treated A549 cells. Compared to the control group, LPS treatment significantly reduced cell viability (Figs. 3a, b, and S3) and induced monolayer damage, increasing cell permeability to FITC–dextran levels (Fig. 3d). Co-treatment with C-MEVs or P-MEVs effectively suppressed the cytotoxicity and epithelial barrier dysfunction in LPS-treated A549 cells in a dose-dependent manner, with P-MEVs showing a more pronounced effect compared to C-MEVs (Fig. 3a–d). These results suggest that inflammatory cytokine-primed MSC-EVs exert superior protective effects against LPS-induced damage compared to unstimulated MSC-EVs. In conclusion, the stimulation of hADMSCs using inflammatory cytokines produces EVs that exert protective effects against LPS-induced inflammation and permeability damage.

### P-MEVs alleviate inflammation in an LPS-induced ALI mouse model

**Fig. 4:**
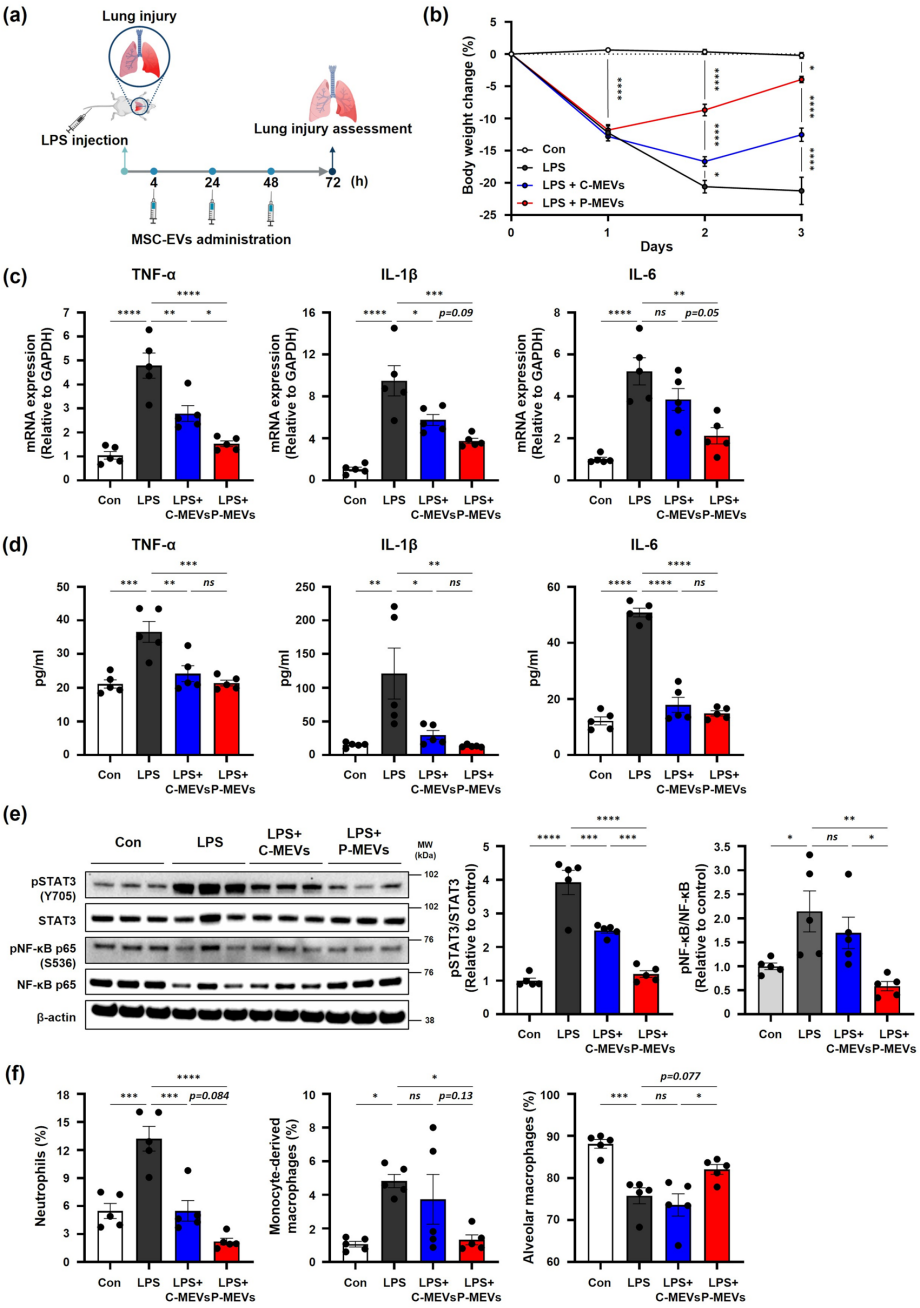
P-MEVs alleviate lung inflammation in a mouse model of LPS-induced ARDS C57BL/6 mice were challenged with LPS (5 mg/kg, intravenously) and then received daily injections of PBS or MSC-EVs (6 × 10^9^ particles, intravenously). After 72 h, the mice were sacrificed and subjected to functional analysis. (**a**) Experimental scheme for establishing an LPS-induced ALI mouse model and administration of MSC-EVs. Created using BioRender.com. (**b**) Daily monitoring of mouse body weight changes. Values are calculated as a percentage of body weight from day 0. *n =* 4–5. (**c**) qRT-PCR analysis of relative mRNA expression levels of *TNF-α*, *IL-1β*, and *IL-6* in the lung tissues. *n =* 5. (**d**) ELISA analysis of TNF-α, IL-1β, and IL-6 concentrations in the lung tissues. *n =* 5. (**e**) Western blot analysis of lung homogenate (left, Full-length blots are presented in Supplementary Figure S10). Phosphorylated bands were normalized to the respective total bands and are shown as fold changes relative to the control. *n =* 5. (**f**) Flow cytometric analysis of neutrophils (CD11b + Ly6G+), monocyte-derived macrophages (Siglec F- CD11b + on CD11c + F4/80+), and alveolar macrophages (Siglec F + CD11b- on CD11c + F4/80+) accumulation in lung tissue. *n =* 5. Con, control mice with PBS injections; LPS, mice with LPS injection only; LPS + C-MEVs, mice with LPS and control MSC-EVs injections; LPS + P-MEVs, mice with LPS and primed MSC-EVs injections. Data are presented as the mean ± SEM, analyzed by two-way ANOVA for (**b**) and analyzed with a one-way ANOVA for (**c**), (**d**), (**e**), and (**f**), followed by the Holm–Sidak multiple comparison test. Statistical differences in post hoc tests are indicated as *ns* = not significant, *p < 0.05, **p < 0.01, ***p < 0.001, and ****p < 0.0001

LPS instillation into the lung is a widely used model to induce ALI in animals and shares key pathologic features with ALI, such as alveolar space thickening and elevated levels of inflammatory cytokines [32]. To confirm the therapeutic efficacy of P-MEVs in ALI in vivo, we established a mouse model of ALI by intravenous injection of 5 mg/kg LPS. Subsequently, the mice received daily tail vein injections of MSC-EVs (Fig. 4a). This intravenous administration route was chosen as it is the most established and reproducible method for such preclinical models, ensuring reliable assessment of therapeutic potential [33]. After LPS treatment, mice showed a gradual decrease in body weight. However, the administration of either C-MEVs or P-MEVs significantly attenuated this body weight loss, and the effect was particularly remarkable following P-MEVs administration (Fig. 4b). After three days of LPS treatment, lung tissues were harvested and evaluated for inflammatory response and ALI. First, we analyzed the mRNA expression levels of proinflammatory cytokines, including TNF-α, IL-1β, and IL-6 in the lung tissue. LPS treatment significantly upregulated the mRNA expression of these cytokines compared to the control group. Notably, mice injected with either C-MEVs or P-MEVs showed a decrease in the mRNA expression of inflammatory cytokines compared to those treated with LPS alone, with P-MEVs demonstrating superior efficacy over C-MEVs (Fig. 4c). We further investigated the effect of MSC-EVs on protein levels of proinflammatory cytokines using ELISA. LPS treatment significantly increased the secretion of proinflammatory cytokines in the lung tissue of mice, which was ameliorated by administering either C-MEVs or P-MEVs (Fig. 4d). Western blot analysis of lung homogenates revealed a significant increase in the phosphorylation of STAT3 and NF-κB, key proinflammatory signaling pathways, in LPS-challenged mice. In contrast, administration of either C-MEVs or P-MEVs attenuated these inflammatory signaling pathways, with P-MEV administration showing enhanced efficacy (Fig. 4e). Having established that P-MEVs mitigate both cytokine production and inflammatory signaling, we next explored how these treatments affect immune cell infiltration in the lung. Accordingly, we performed a flow cytometric analysis of neutrophils, monocyte-derived macrophages, and alveolar macrophages on lung homogenates. LPS-treated mice showed significantly increased recruitment of neutrophils and monocyte-derived macrophages. Conversely, administration of either C-MEVs or P-MEVs decreased the amount of these cells (Figs. 4f and S4). In addition, we also observed that administration of either C-MEVs or P-MEVs to LPS-treated mice increased the proportion of alveolar macrophages, the subset necessary for tissue homeostasis and repair during ALI. Notably, the administration of P-MEVs had a greater impact on reducing ALI than the administration of C-MEVs, consistent with previous results (Figs. 4f and S4). These results demonstrate that P-MEVs have superior efficacy to C-MEVs against LPS-induced lung inflammation, highlighting their potential as an advanced therapeutic approach for ALI.

### P-MEVs attenuate LPS-induced lung injury, pulmonary edema, and vascular leakage

**Fig. 5:**
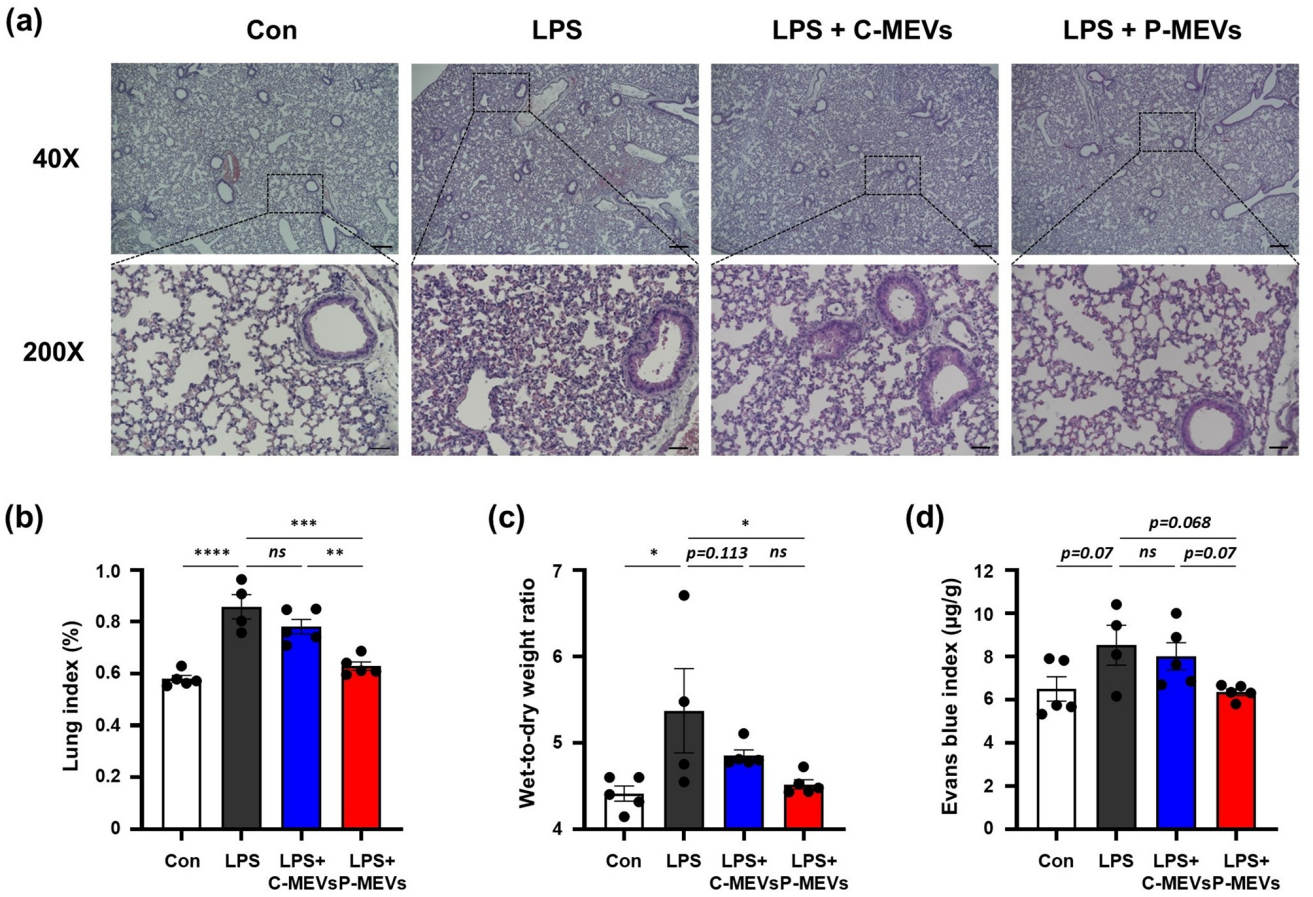
P-MEVs attenuate LPS-induced lung injury, pulmonary edema, and vascular leakage C57BL/6 mice were administered LPS (5 mg/kg, intravenously) and received daily injections of PBS or MSC-EVs (6 × 10^9^ particles, intravenously). After 72 h, the mice were sacrificed and subjected to functional analysis. (**a**) Representative H&E staining of lung tissue. Black scale bars represent 250 μm at 40x and 50 μm at 200x. (**b**) Evaluated lung index (%; lung weight (g)/body weight (g) x 100). *n =* 4–5. (**c**) Lung W/D ratio for pulmonary edema evaluations. *n =* 4–5. (**d**) Evans blue index (µg/g) for assessing lung permeability. Mice received a tail vein injection of 1% Evans blue 2 h before euthanasia. *n =* 4–5. One mouse in the LPS group died before data collection could be performed and was excluded from the study. Con, control mice with PBS injections; LPS, mice with LPS injection only; LPS + C-MEVs, mice with LPS and control MSC-EVs injections; LPS + P-MEVs, mice with LPS and primed MSC-EVs injections. Data are presented as the mean ± SEM, analyzed by one-way ANOVA, followed by the Holm–Sidak multiple comparison test. Statistical differences in post hoc tests are indicated as *ns* = not significant, *p < 0.05, **p < 0.01, ***p < 0.001, and ****p < 0.0001

Improving key pathological features such as lung injury, pulmonary edema, and vascular leakage is critical in treating ALI, as these indicators are directly related to disease severity and patient outcomes [34, 35]. First, we evaluated the effects of administering P-MEVs on LPS-induced lung injury, pulmonary edema, and vascular leakage. Histological analysis of lung sections revealed substantial lung damage in LPS-treated mice, including immune cell infiltration and increased alveolar wall thickness (Fig. 5a). However, administering P-MEVs notably attenuated this damage, as evidenced by reduced immune cell infiltration and alveolar wall thickening (Fig. 5a). Administering C-MEVs showed similar improvements, though to a lesser extent (Fig. 5a). To further evaluate the effect of administering MSC-EVs on LPS-induced pulmonary edema, we measured the lung index and W/D ratio of each group. Compared to the control group, both parameters were significantly increased in the LPS-treated group. However, administering either C-MEVs or P-MEVs markedly reduced pulmonary edema in LPS-challenged mice (Fig. 5b, c). We measured the leak index using Evans blue to assess the severity of lung vascular leakage. Evans blue leakage increased in the LPS-treated group compared to the normal group. However, administering either C-MEVs or P-MEVs reduced capillary permeability compared to the LPS-only treated group (Fig. 5d). Interestingly, P-MEV administration was associated with a further improvement in pulmonary edema and vascular leakage compared to C-MEV administration (Fig. 5b–d). Collectively, P-MEVs showed a superior ability to alleviate lung injury, reduce pulmonary edema, and improve vascular integrity in LPS-treated mice, demonstrating greater therapeutic potential than C-MEVs.

### P-MEVs ameliorate the SARS-CoV-2-induced damage in SARS-CoV-2-infected cells

**Fig. 6:**
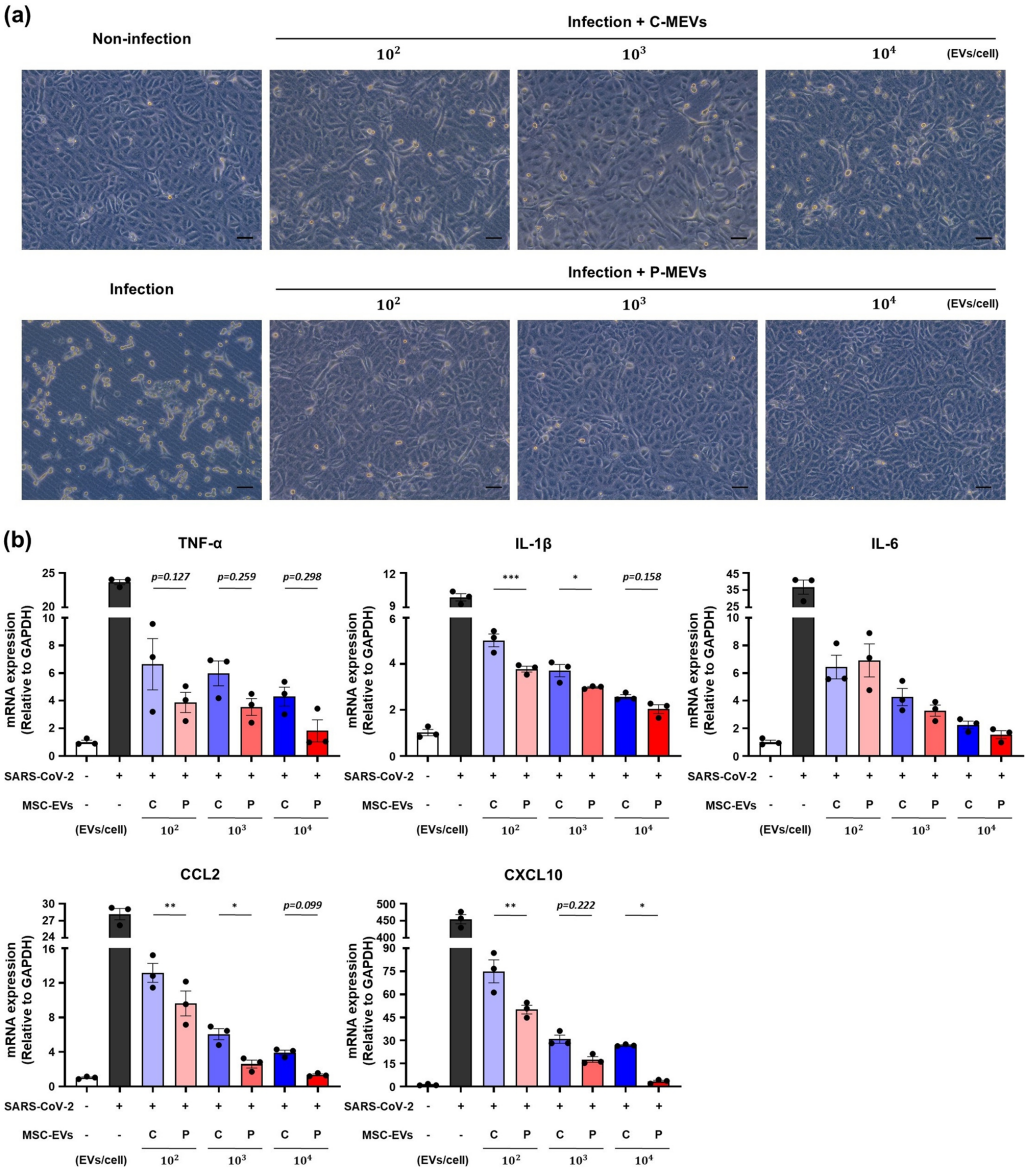
P-MEVs ameliorate the SARS-CoV-2 infection-induced damage in vitro Vero E6 cells were infected with SARS-CoV-2 (MOI = 0.003) for 1 h, followed by exposure to C-MEVs or P-MEVs for 48 h. (**a**) Representative images of CPE were observed under a microscope. Black scale bars represent 25 μm. (**b**) qRT-PCR analysis of relative mRNA expression levels of *TNF-α*, *IL-1β*, *IL-6*, *CCL2*, and *CXCL10*. *n =* 3. C-MEVs, control MSC-EVs; P-MEVs, primed MSC-EVs; C, control; P, primed. Data are presented as the mean ± SEM, analyzed by one-way ANOVA, followed by the Holm–Sidak multiple comparison test. Statistical differences in post hoc tests are indicated as *p < 0.05, **p < 0.01, and ***p < 0.001

To explore whether P-MEVs could alleviate the inflammatory responses associated with coronavirus disease 2019 (COVID-19)-induced damage, we examined the anti-inflammatory efficacy of MSC-EVs in SARS-CoV-2-infected Vero E6 cells. The Vero E6 cell line, derived from African green monkey kidney epithelial cells, is commonly used to study SARS-CoV-2 due to its high expression of ACE2 receptors, which are critical for viral entry [36]. SARS-CoV-2 infection triggers inflammatory responses, increasing proinflammatory cytokines and chemokines [37, 38]. We assessed the cytotoxicity of MSC-EVs on Vero E6 cells by administering different concentrations of C-MEVs or P-MEVs. Both C-MEVs and P-MEVs were observed to be non-toxic, even at high doses (Figure S6). Vero E6 cells were then infected with SARS-CoV-2 at 0.003 MOI and incubated for 1 h. MSC-EVs were treated after the virus was removed. After 48 h, CPE was evaluated by microscopy. SARS-CoV-2-infected cells showed severe CPE, but treatment with C-MEVs or P-MEVs effectively suppressed the viral toxicity in a dose-dependent manner (Fig. 6a). Furthermore, we investigated the influence of MSC-EVs on inflammation by measuring changes in mRNA expression levels of inflammatory markers associated with COVID-19 severity, including *TNF-α*, *IL-1β*, *IL-6*, *CCL2*, and *CXCL10*. SARS-CoV-2 infection strongly upregulated the mRNA expression of the aforementioned inflammatory cytokines and chemokines. However, treatment with C-MEVs or P-MEVs effectively suppressed the SARS-CoV-2-induced inflammation in a dose-dependent manner, with enhanced effects observed when MSC-EVs were primed with inflammatory cytokines (Fig. 6b). Taken together, these findings suggest that P-MEVs are promising candidates for mitigating SARS-CoV-2-induced inflammation, as they more effectively reduce viral cytotoxicity and inflammatory responses than C-MEVs, potentially pointing toward future applications in COVID-19-related lung injury.

### MiRNAs enriched in P-MEVs suppress LPS-induced inflammation in macrophages and enhance therapeutic potential against ALI

**Fig. 7:**
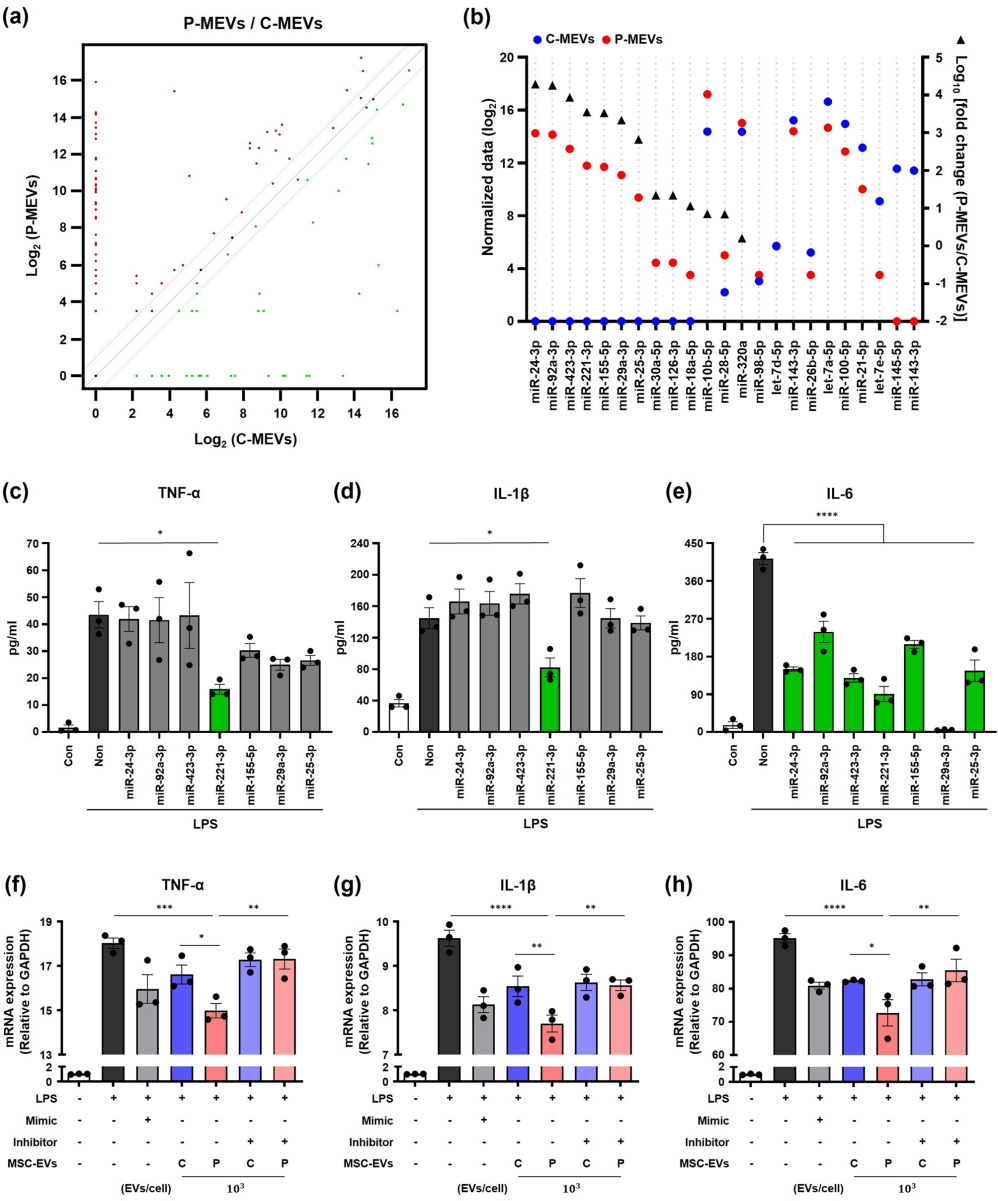
P-MEV-enriched miRNAs suppress LPS-induced inflammation in macrophages (**a**) Scatter plot of differential expression of miRNAs between C-MEVs (𝑥-axis) and P-MEVs (𝑦-axis). The red and green lines represent the threshold for a 2-fold increase and decrease in miRNA levels in P-MEVs compared to C-MEVs, respectively. (**b**) The normalized data of C-MEVs (blue circle) and P-MEVs (red circle) are shown on the left 𝑦-axis with their corresponding fold change (black triangle on the right 𝑦-axis) of 23 miRNAs in negative regulation of inflammation. (c–e) Protein expressions of TNF-α (**c**), IL-1β (**d**), and IL-6 (**e**) in THP-1 cells at 24 h after transfection with each of the seven miRNA candidates (100 nM), followed by treatment with 5 µg/mL LPS using ELISA. *n =* 3. (**f-h**) qRT-PCR analysis of relative mRNA expression levels of TNF-α (**c**), IL-1β (**d**), and IL-6 (**e**) in THP-1 cells 6 h post LPS exposure (5 µg/mL). Cells were transfected with a miR-221-3p mimic or miR-221-3p inhibitor or Control (100 nM) for 24 h prior to LPS and EV treatment. *n =* 3. C-MEVs, control MSC-EVs; P-MEVs, primed MSC-EVs. Data are presented as the mean ± SEM, analyzed by one-way ANOVA, followed by the Holm–Sidak multiple comparison test. Statistical differences in post hoc tests are indicated as *p < 0.05 and ****p < 0.0001

It is well-established that miRNAs mediate the therapeutic effects of MSC-EVs in ALI [39, 40]. Therefore, we compared overall miRNA profiles between C-MEVs and P-MEVs using miRNA sequencing (miRseq) to investigate the potential contributing factors that enhanced the efficacy of P-MEVs in ALI treatment. A total of 193 miRNAs were found in C-MEVs and P-MEVs, with 101 miRNAs in P-MEVs showing more than twice the expression level of those in C-MEVs (Fig. 7a). To explore the genes targeted by the top 10 miRNAs highly expressed in P-MEVs (expression changes by P-MEVs were greater than 2-fold compared to C-MEVs), we further conducted GO and KEGG pathway analyses (Figure S7a, b). The GO analysis revealed that the identified miRNAs were predominantly associated with transcriptional regulation, protein binding, and DNA-binding transcription factor activity (Figure S7a). KEGG analysis highlighted pathways such as proteoglycans in cancer and the mitogenactivated protein kinase signaling pathway (Figure S7b). Notably, miRseq analysis revealed that both C-MEVs and P-MEVs contained miRNAs critical for regulating the immune response, promoting tissue regeneration, and, importantly, suppressing inflammation (Figure S8a–c). These key miRNAs, detected in both groups, highlight the fundamental role of miRNAs in modulating inflammatory processes and enhancing the therapeutic potential of MSC-EVs in ALI treatment. Based on these miRseq data, we hypothesized that miRNAs associated with negatively regulating inflammation suppress the LPS-induced inflammation. Among the 23 miRNAs involved in negatively regulating inflammation, seven whose expression was increased by more than 600-fold in P-MEVs compared to C-MEVs were selected to assess their influence on the secretion of proinflammatory cytokines in macrophages (Fig. 7b). We transfected THP-1 cells with each miRNA mimic and evaluated the reduction in the LPS-induced secretion of proinflammatory cytokines. Compared to the control group, stimulation with 5 µg/mL LPS resulted in a significant increase in the secretion of TNF-α, IL-1β, and IL-6 in non-transfected THP-1 cells (Fig. 7c–e). Conversely, miR-221-3p downregulated the protein levels of TNF-α and IL-1β in LPS-treated THP-1 cells (Fig. 7c, d). Interestingly, all miRNAs (miR-24-3p, miR-92a-3p, miR-423-3p, miR-221-3p, miR-155-5p, miR-29a-3p, and miR-25-3p) significantly reduced IL-6 secretion in LPS-treated THP-1 cells (Fig. 7e). To further validate that miR-221-3p is a key mediator of the enhanced therapeutic effects of P-MEVs, we conducted a critical loss-of-function experiment. We transfected THP-1 cells with a specific miR-221-3p inhibitor prior to treatment with P-MEVs under LPS-induced inflammatory conditions. Notably, the functional inhibition of miR-221-3p in recipient cells markedly abrogated the superior anti-inflammatory capacity of P-MEVs. The ability of P-MEVs to suppress the production of TNF-α, IL-1β, and IL-6 was significantly reversed, confirming that their therapeutic effect is critically dependent on miR-221-3p activity (Fig. 7f–h). In conclusion, these findings suggest that the enhanced efficacy of P-MEVs in ALI treatment may be due to the expression of specific miRNAs and their anti-inflammatory effects.

## Discussion

The primary objective of this study was to evaluate the extent to which the immunomodulatory and tissue-regenerative capacities of EVs derived from IFN-γ/TNF-α-primed hADMSCs could be enhanced relative to unprimed EVs in an LPS-induced ALI model. We further aimed to determine whether P-MEVs could suppress inflammatory responses in an in vitro SARS-CoV-2 infection model, thereby suggesting a potential extension to virus-induced lung injury. Previous research has indicated that MSC-EVs exert therapeutic effects in severe inflammatory diseases, including ALI and ARDS. However, unprimed EVs frequently exhibit limited immunomodulatory capacity, rendering them less effective against the intense inflammatory environment characterizing severe conditions [17–19]. In this study, we administered C-MEVs and P-MEVs under identical conditions in an LPS-induced ALI model and observed significantly reduced expression of proinflammatory cytokines, along with marked improvements in lung tissue injury indices and pulmonary edema in the P-MEV group. These findings strongly support the premise that priming MSCs with IFN-γ and TNF-α substantially enhances the immunomodulatory and tissue-regenerative properties of their EVs, clearly demonstrating superior therapeutic efficacy compared to control EVs.

An essential consideration for the clinical translation of any new therapeutic is its safety profile. MSC-EVs inherently offer safety advantages over cell-based therapies, such as avoiding the risks of embolism [41]. Importantly, our findings are consistent with existing literature demonstrating the safety of primed EVs. A key preclinical study showed that while inflammatory cytokine-primed MSCs could be lethal in a severe disease model, the EVs derived from these same cells were well-tolerated and therapeutically effective [42]. This supports the notion that priming enhances therapeutic function without conferring the risks of the parent cells. In our study, P-MEVs exhibited no cytotoxicity in vitro and were well-tolerated in our ALI model, as evidenced by the significant attenuation of body weight loss in treated mice. Taken together, this collective evidence strongly supports the preclinical safety of P-MEVs as a promising therapeutic agent for ALI.

In line with widely accepted preclinical criteria for ALI models, we evaluated multiple domains that reflect key aspects of lung injury pathophysiology. Specifically, our study incorporated: (i) histological assessment of alveolar injury, (ii) quantification of alveolar-capillary barrier disruption, and (iii) evaluation of inflammation. These measurements fulfill three of the four core domains outlined in the updated experimental ALI guidelines, which recommend reporting at least one relevant parameter from each domain to ensure pathophysiological relevance [43]. Although we did not directly assess mechanical indices such as lung compliance or airway resistance, these measurements typically require specialized ventilator systems and invasive procedures—such as tracheal intubation under anesthesia—that were incompatible with our survival-based protocol. Instead, we adopted surrogate markers that are widely accepted as indirect indicators of pulmonary function and gas exchange capacity in murine ALI models [44]. Taken together, our findings demonstrate that P-MEVs not only modulate inflammation but also alleviate functional impairments in lung structure and barrier integrity, capturing key features of ALI progression and recovery.

Viral lung injury is likewise characterized by potent inflammatory responses and tissue destruction, highlighting a substantial therapeutic gap [45, 46]. SARS-CoV-2, which causes COVID-19, has led to widespread virus-induced lung injuries worldwide due to its high infectivity and mortality rates [2, 3]. In this study, to explore whether the potent anti-inflammatory effects of P-MEVs extend to a viral context, we applied them to an in vitro SARS-CoV-2-infected cell model. We observed more pronounced anti-inflammatory effects—manifested by reduced expression and secretion of inflammatory mediators—and enhanced cell viability relative to the C-MEV group. This finding suggests that primed EVs may suppress excessive immune reactions even under viral conditions, paving the way for a cell-free therapeutic strategy against COVID-19 and related virus-induced ALI. It is important to note, however, that this experiment was designed as a proof-of-concept to assess the anti-inflammatory potential of P-MEVs, and a comprehensive evaluation of direct antiviral activity, such as measuring viral replication, was beyond the scope of the present work. Therefore, future studies including in vivo viral challenge models and detailed virological assessments are necessary to fully establish their therapeutic potential in this context.

From a mechanistic standpoint, multiple studies have proposed that primed MSCs upregulate anti-inflammatory mediators such as COX-2, IDO, and TSG-6 and increase the loading of specific miRNAs. The present results also suggest that P-MEVs contain higher levels of these anti-inflammatory substances and miRNAs than C-MEVs, thereby more effectively inhibiting NF-κB, STAT, and other inflammatory signaling pathways. Notably, we observed a pronounced elevation of miR-221-3p in P-MEVs, and cells treated with a miR-221-3p mimic under LPS-induced injury displayed a robust anti-inflammatory response, as evidenced by reduced TNF-α, IL-1β, and IL-6 levels. Crucially, our loss-of-function experiment provides direct evidence for this proposed mechanism. By inhibiting miR-221-3p in recipient macrophages, we demonstrated that the enhanced anti-inflammatory effects of P-MEVs were significantly diminished. This finding confirms that miR-221-3p is not merely a correlational marker but a key functional mediator of the heightened therapeutic efficacy of P-MEVs. Previous research has demonstrated that miR-221-3p plays a pivotal role in modulating inflammatory pathways and immune responses in lung disease models, particularly by suppressing cell apoptosis in conditions such as sepsis-induced ALI and chronic obstructive pulmonary disease [47, 48]. Moreover, miR-221-3p has been identified as a major regulator of inflammatory responses in severe COVID-19 patients, suggesting its potential for controlling the excessive inflammation associated with COVID-19-induced ALI [49]. Collectively, these findings indicate that miR-221-3p may serve as a promising therapeutic candidate for mitigating inflammation in both acute and chronic lung injury models, particularly in COVID-19-induced damage. By effectively downregulating proinflammatory cytokines, P-MEVs enriched with miR-221-3p could be employed as a potent intervention for ALI and other serious inflammatory disorders, thus opening new avenues for miRNA-based therapies targeting both pulmonary and systemic inflammatory diseases.

Beyond the enhanced efficacy observed in ALI and SARS-CoV-2–induced models, our findings have broader implications for treating other inflammatory and immune-mediated conditions. The ability of inflammatory cytokine priming to selectively enrich EVs with therapeutic miRNAs without compromising EV integrity or yield opens the door to tailoring EV cargo to specific disease contexts, potentially enabling personalized cell-free therapies. Future studies should explore the utility of primed EVs in other inflammatory disorders, such as sepsis or autoimmune diseases, and assess the benefits of combining EV-based therapy with existing pharmacological treatments. Such combination strategies could further improve therapeutic outcomes by simultaneously modulating multiple facets of the immune response. Ultimately, these efforts will be crucial for advancing primed MSC-EV therapies from bench to bedside.

Although our results clearly demonstrate that inflammatory cytokine priming enhances the therapeutic efficacy of MSC-EVs in mitigating ALI, further investigation is warranted. A limitation of our study is the lack of direct in situ evidence to precisely identify which cell populations within the lung tissue are responsible for the reduced inflammatory signaling we observed. Future studies should employ more advanced techniques to address this. For instance, multiplex immunofluorescence (IF) for pSTAT3 could be used to visually confirm these cell-specific effects directly within the lung tissue. Furthermore, intracellular flow cytometry would allow for the precise quantification of changes in pSTAT3 levels within distinct cell populations, thereby fully dissecting the mechanisms of P-MEVs in vivo. Furthermore, it is important to optimize priming conditions and elucidate the mechanisms underlying selective miRNA enrichment. In particular, the molecular basis for the preferential packaging of key regulatory miRNAs, such as miR-221-3p, remains incompletely understood. Future studies should also focus on refining priming protocols and assessing the long-term stability and biodistribution of primed EVs in vivo. Moreover, employing more clinically relevant models—especially those that recapitulate the complex inflammatory milieu of COVID-19-induced ARDS—will be essential for translating these findings into effective cell-free therapies. Such efforts could lay the groundwork for combining MSC-EV treatment with other therapeutic strategies, thereby further enhancing clinical outcomes in patients with severe pulmonary diseases.

## Conclusion

In conclusion, P-MEVs exhibited significantly enhanced immunomodulatory and tissue-regenerative effects in an ALI model compared to C-MEVs, while also demonstrating anti-inflammatory capabilities in a SARS-CoV-2-infected cell model, suggesting their potential extension to virus-induced lung injury. These findings support the notion that priming can substantially enhance the therapeutic efficacy of EVs as a cell-free treatment option and indicate that P-MEVs could be applied to various severe respiratory diseases, including virus-mediated ALI. With further assessment in large-animal studies and clinical trials, primed EVs may emerge as an innovative strategy to address current therapeutic gaps in managing severe lung injuries.

## Supplementary Information

Below is the link to the electronic supplementary material.

Supplementary Material 1

## Data Availability

The datasets generated and/or analysed during the current study are available in the NCBI Gene Expression Omnibus (GEO) repository, under accession number GSE293280. The data can be accssed at [https://www.ncbi.nlm.nih.gov/geo/query/acc.cgi?acc=GSE293280].

## References

[1] Rubenfeld GD, Caldwell E, Peabody E, Weaver J, Martin DP, Neff M, et al. Incidence and outcomes of acute lung injury. New Engl J Med. 2005;353(16):1685–93. 16236739 10.1056/NEJMoa050333

[2] Henry BM. COVID-19, ECMO, and lymphopenia: a word of caution. Lancet Respir Med. 2020;8(4):e24. 32178774 10.1016/S2213-2600(20)30119-3 PMC7118650

[3] Muralidar S, Ambi SV, Sekaran S, Krishnan UM. The emergence of COVID-19 as a global pandemic: Understanding the epidemiology, immune response and potential therapeutic targets of SARS-CoV-2. Biochimie. 2020;179:85–100. 32971147 10.1016/j.biochi.2020.09.018 PMC7505773

[4] Ware LB. The acute respiratory distress syndrome (342, Pg 1334, 2000). New Engl J Med. 2000;343(7):520. 10.1056/nejm200008173430722 10944572

[5] Confalonieri M, Salton F, Fabiano F. Acute respiratory distress syndrome. Eur Respir Rev. 2017;26(144). 10.1183/16000617.0116-2016 PMC9488505 28446599

[6] Matthay MA, Zemans RL, Zimmerman GA, Arabi YM, Beitler JR, Mercat A et al. Acute respiratory distress syndrome. Nat Rev Dis Primers. 2019;5. 10.1038/s41572-019-0069-0 PMC6709677 30872586

[7] Mokra D, Mikolka P, Kosutova P, Mokry J. Corticosteroids in acute lung injury: the dilemma continues. Int J Mol Sci. 2019;20(19). 10.3390/ijms20194765 PMC6801694 31557974

[8] Spinelli E, Mauri T, Beitler JR, Pesenti A, Brodie D. Respiratory drive in the acute respiratory distress syndrome: pathophysiology, monitoring, and therapeutic interventions. Intens Care Med. 2020;46(4):606–18. 10.1007/s00134-020-05942-6 PMC7224136 32016537

[9] Bain W, Yang HP, Shah FA, Suber T, Drohan C, Al-Yousif N, et al. COVID-19 versus Non-COVID-19 acute respiratory distress syndrome comparison of demographics, physiologic parameters, inflammatory biomarkers, and clinical outcomes. Ann Am Thorac Soc. 2021;18(7):1202–10. 33544045 10.1513/AnnalsATS.202008-1026OC PMC8328355

[10] Horie S, McNicholas B, Rezoagli E, Pham T, Curley G, McAuley D, et al. Emerging Pharmacological therapies for ARDS: COVID-19 and beyond. Intens Care Med. 2020;46(12):2265–83. 10.1007/s00134-020-06141-z PMC7352097 32654006

[11] Chen TS, Lai RC, Lee MM, Choo ABH, Lee CN, Lim SK. Mesenchymal stem cell secretes microparticles enriched in pre-microRNAs. Nucleic Acids Res. 2010;38(1):215–24. 19850715 10.1093/nar/gkp857 PMC2800221

[12] Lai RC, Tan SS, Teh BJ, Sze SK, Arslan F, de Kleijn DP, et al. Proteolytic potential of the MSC exosome proteome: implications for an exosome-Mediated delivery of therapeutic proteasome. Int J Proteom. 2012;2012:971907. 10.1155/2012/971907 PMC3407643 22852084

[13] Lee C, Mitsialis SA, Aslam M, Vitali SH, Vergadi E, Konstantinou G, et al. Exosomes mediate the cytoprotective action of mesenchymal stromal cells on hypoxia-induced pulmonary hypertension. Circulation. 2012;126(22):2601–11. 23114789 10.1161/CIRCULATIONAHA.112.114173 PMC3979353

[14] Zhao RJ, Wang LN, Wang T, Xian PP, Wang HK, Long QF. Inhalation of MSC-EVs is a noninvasive strategy for ameliorating acute lung injury. J Control Release. 2022;345:214–30. 35307508 10.1016/j.jconrel.2022.03.025

[15] Askenase PW. COVID-19 therapy with mesenchymal stromal cells (MSC) and convalescent plasma must consider exosome involvement: do the exosomes in convalescent plasma antagonize the weak immune antibodies? J Extracell Vesicles. 2020;10(1). 10.1002/jev2.12004 PMC7710130 33304473

[16] Tieu A, Hu K, Gnyra C, Montroy J, Fergusson DA, Allan DS et al. Mesenchymal stromal cell extracellular vesicles as therapy for acute and chronic respiratory diseases: A meta-analysis. J Extracell Vesicles. 2021;10(12). 10.1002/jev2.12141 PMC8485337 34596349

[17] Galipeau J, Sensébé L. Mesenchymal stromal cells: clinical challenges and therapeutic opportunities. Cell Stem Cell. 2018;22(6):824–33. 29859173 10.1016/j.stem.2018.05.004 PMC6434696

[18] Lener T, Gimona M, Aigner L, Borger V, Buzas E, Camussi G, et al. Applying extracellular vesicles based therapeutics in clinical trials - an ISEV position paper. J Extracell Vesicles. 2015;4:30087. 26725829 10.3402/jev.v4.30087 PMC4698466

[19] Homma K, Bazhanov N, Hashimoto K, Shimizu M, Heathman T, Hao Q et al. Mesenchymal stem cell-derived exosomes for treatment of sepsis. Front Immunol. 2023;14. 10.3389/fimmu.2023.1136964 PMC10169690 37180159

[20] Ren GW, Zhang LY, Zhao X, Xu GW, Zhang YY, Roberts AI, et al. Mesenchymal stem cell-mediated immunosuppression occurs via concerted action of chemokines and nitric oxide. Cell Stem Cell. 2008;2(2):141–50. 18371435 10.1016/j.stem.2007.11.014

[21] Losurdo M, Pedrazzoli M, D’Agostino C, Elia CA, Massenzio F, Lonati E, et al. Intranasal delivery of mesenchymal stem cell-derived extracellular vesicles exerts Immunomodulatory and neuroprotective effects in a 3xTg model of alzheimer’s disease. Stem Cell Transl Med. 2020;9(9):1068–84. 10.1002/sctm.19-0327 PMC7445021 32496649

[22] Song WJ, Li Q, Ryu MO, Ahn JO, Bhang DH, Jung YC et al. TSG-6 secreted by human adipose Tissue-derived mesenchymal stem cells ameliorates DSS-induced colitis by inducing M2 macrophage polarization in mice. Sci Rep-Uk. 2017;7. 10.1038/s41598-017-04766-7 PMC5507867 28701721

[23] Zhang Q, Fu L, Liang Y, Guo Z, Wang L, Ma C, et al. Exosomes originating from MSCs stimulated with TGF-beta and IFN-gamma promote Treg differentiation. J Cell Physiol. 2018;233(9):6832–40. 29336475 10.1002/jcp.26436 PMC13482522

[24] Kang K, Ma RL, Cai WF, Huang W, Paul C, Liang JL et al. Exosomes Secreted from CXCR4 Overexpressing Mesenchymal Stem Cells Promote Cardioprotection via Akt Signaling Pathway following Myocardial Infarction. Stem Cells Int. 2015;2015. 10.1155/2015/659890 PMC4436515 26074976

[25] Krampera M, Galipeau J, Shi YF, Tarte K, Sensebe L, Isct. Immunological characterization of multipotent mesenchymal stromal cells-The international society for cellular therapy (ISCT) working proposal. Cytotherapy. 2013;15(9):1054–61. 23602578 10.1016/j.jcyt.2013.02.010

[26] Bae JH, Lee CH, Jung DKY, Yea K, Song BJ, Lee HK et al. Extracellular vesicle isolation and counting system (EVics) based on simultaneous tandem tangential flow filtration and large field-of-view light scattering. J Extracell Vesicles. 2024;13(7). 10.1002/jev2.12479 PMC11231039 38978321

[27] Welsh JA, Goberdhan DCI, O’Driscoll L, Buzas EI, Blenkiron C, Bussolati B et al. Minimal information for studies of extracellular vesicles (MISEV2023): from basic to advanced approaches. J Extracell Vesicles. 2024;13(2). 10.1002/jev2.12404 PMC10850029 38326288

[28] Tastsoglou S, Skoufos G, Miliotis M, Karagkouni D, Koutsoukos I, Karavangeli A, et al. DIANA-miRPath v4.0: expanding target-based MiRNA functional analysis in cell-type and tissue contexts. Nucleic Acids Res. 2023;51(W1):W154–9. 37260078 10.1093/nar/gkad431 PMC10320185

[29] Mantovani A, Sica A, Sozzani S, Allavena P, Vecchi A, Locati M. The chemokine system in diverse forms of macrophage activation and polarization. Trends Immunol. 2004;25(12):677–86. 15530839 10.1016/j.it.2004.09.015

[30] Munford RS, Varley AW. Shield as signal: lipopolysaccharides and the evolution of immunity to Gram-negative bacteria. Plos Pathog. 2006;2(6):467–71. 10.1371/journal.ppat.0020067 PMC1483240 16846256

[31] Beutler B, Rietschel ET. Innate immune sensing and its roots: the story of endotoxin. Nat Rev Immunol. 2003;3(2):169–76. 12563300 10.1038/nri1004

[32] Matute-Bello G, Frevert CW, Martin TR. Animal models of acute lung injury. Am J Physiol-Lung C. 2008;295(3):L379–99. 10.1152/ajplung.00010.2008 PMC2536793 18621912

[33] Tieu A, Hu K, Gnyra C, Montroy J, Fergusson DA, Allan DS, et al. Mesenchymal stromal cell extracellular vesicles as therapy for acute and chronic respiratory diseases: A meta-analysis. J Extracell Vesicles. 2021;10(12):e12141. 34596349 10.1002/jev2.12141 PMC8485337

[34] Matthay MA, Ware LB, Zimmerman GA. The acute respiratory distress syndrome. J Clin Invest. 2012;122(8):2731–40. 22850883 10.1172/JCI60331 PMC3408735

[35] Thompson BT, Chambers RC, Liu KD. Acute respiratory distress syndrome. New Engl J Med. 2017;377(6):562–72. 28792873 10.1056/NEJMra1608077

[36] Rosa RB, Dantas WM, do Nascimento JCF, da Silva MV, de Oliveira RN, Pena LJ. In Vitro and in vivo models for studying SARS-CoV-2, the etiological agent responsible for COVID-19 pandemic. Viruses-Basel. 2021;13(3). 10.3390/v13030379 PMC7997194 33673614

[37] Lucas C, Wong P, Klein J, Castro TBR, Silva J, Sundaram M, et al. Longitudinal analyses reveal immunological misfiring in severe COVID-19. Nature. 2020;584(7821):463–. 32717743 10.1038/s41586-020-2588-y PMC7477538

[38] Tay MZ, Poh CM, Rénia L, MacAry PA, Ng LFP. The trinity of COVID-19: immunity, inflammation and intervention. Nat Rev Immunol. 2020;20(6):363–74. 32346093 10.1038/s41577-020-0311-8 PMC7187672

[39] Yi XM, Wei XX, Lv HJ, An YL, Li LJ, Lu PL et al. Exosomes derived from microRNA-30b-3p-overexpressing mesenchymal stem cells protect against lipopolysaccharide-induced acute lung injury by inhibiting SAA3. Exp Cell Res. 2019;383(2). 10.1016/j.yexcr.2019.05.035 31170401

[40] Liang CY, Liu Y, Xu HF, Huang JL, Shen Y, Chen FX et al. Exosomes of human umbilical cord MSCs protect against Hypoxia/Reoxygenation-Induced pyroptosis of cardiomyocytes via the miRNA-100-5p/FOXO3/NLRP3 pathway. Front Bioeng Biotech. 2021;8. 10.3389/fbioe.2020.615850 PMC7844314 33520966

[41] Al-Masawa ME, Elfawy LA, Ng CY, Ng MH, Law JX. Mesenchymal stromal Cell-Derived extracellular vesicles in the management of atopic dermatitis: A scoping review of therapeutic opportunities and challenges. Int J Nanomed. 2025;20:2673–93. 10.2147/IJN.S494574 PMC11890010 40061879

[42] Hackel A, Vollmer S, Bruderek K, Lang S, Brandau S. Immunological priming of mesenchymal stromal/stem cells and their extracellular vesicles augments their therapeutic benefits in experimental graft-versus-host disease via engagement of PD-1 ligands. Front Immunol. 2023;14:1078551. 36875112 10.3389/fimmu.2023.1078551 PMC9978482

[43] Kulkarni HS, Lee JS, Bastarache JA, Kuebler WM, Downey GP, Albaiceta GM, et al. Update on the features and measurements of experimental acute lung injury in animals: an official American thoracic society workshop report. Am J Respir Cell Mol Biol. 2022;66(2):e1–14. 35103557 10.1165/rcmb.2021-0531ST PMC8845128

[44] Matute-Bello G, Downey G, Moore BB, Groshong SD, Matthay MA, Slutsky AS, et al. An official American thoracic society workshop report: features and measurements of experimental acute lung injury in animals. Am J Respir Cell Mol Biol. 2011;44(5):725–38. 21531958 10.1165/rcmb.2009-0210ST PMC7328339

[45] Arentz M, Yim E, Klaff L, Lokhandwala S, Riedo FX, Chong M, et al. Characteristics and outcomes of 21 critically ill patients with COVID-19 in Washington state. Jama-J Am Med Assoc. 2020;323(16):1612–4. 10.1001/jama.2020.4326 PMC7082763 32191259

[46] Zhou F, Yu T, Du RH, Fan GH, Liu Y, Liu ZB, et al. Clinical course and risk factors for mortality of adult inpatients with COVID-19 in wuhan, china: a retrospective cohort study. Lancet. 2020;395(10229):1054–62. 32171076 10.1016/S0140-6736(20)30566-3 PMC7270627

[47] Wang GZ, Ma XX, Huang WC, Wang SH, Lou AN, Wang J et al. Macrophage biomimetic nanoparticle-targeted functional extracellular vesicle micro-RNAs revealed via multiomics analysis alleviate sepsis-induced acute lung injury. J Nanobiotechnol. 2024;22(1). 10.1186/s12951-024-02597-z PMC11194988 38910259

[48] Yang H, Zhang LJ, Wang QD. MicroRNA-221-3p alleviates cell apoptosis and inflammatory response by targeting Cyclin dependent kinase inhibitor 1B in chronic obstructive pulmonary disease. Bioengineered. 2021;12(1):5705–15. 34516316 10.1080/21655979.2021.1967837 PMC8806819

[49] Gaytán-Pacheco N, Ibáñez-Salazar A, Herrera-Van Oostdam AS, Oropeza-Valdez JJ, Magaña-Aquino M, López JA et al. miR-146a, miR-221, and miR-155 are involved in inflammatory immune response in severe COVID-19 patients. Diagnostics. 2023;13(1). 10.3390/diagnostics13010133 PMC9818442 36611425

