# Supplementary material for: Inflammatory cytokine-primed MSC-derived extracellular vesicles ameliorate acute lung injury via enhanced immunomodulation and alveolar repair
Source: Stem Cell Res Ther. 2025 Aug 22;16:450. doi: 10.1186/s13287-025-04576-z (PMC12374373; doi:10.1186/s13287-025-04576-z)
Supplement: Supplementary file 1 — Supplementary Material 1 [file 13287_2025_4576_MOESM1_ESM.docx]

Supporting Information for

**Inflammatory cytokine-primed MSC-derived extracellular vesicles: a novel approach to combat COVID-19-induced ARDS**

Jongwon Jeong^1^**^†^**, Jun-Kook Park^1^**^†^**, Jiwon Shin^1^, Inseong Jung^1^, Hyun-Woo Kim^2^, Anyeseu Park^3^, Hanchae Cho^4^, Sung-Min Kang^5^, Sanghee Shin^1^, Eunju Park^1^, Jisuk Kim^1^, Soojeong Noh^1^, Yongdeok Ahn^6^, Do-Kyun Kim^2^, Jeong Yoon Lee^3^, Daeha Seo^6^, Moon-Chang Baek^5,*^, and Kyungmoo Yea^1,7,*^

^1^Department of New Biology, Daegu Gyeongbuk Institute of Science and Technology (DGIST), Daegu, 42988, Republic of Korea

^2^Korea Zoonosis Research Institute, Jeonbuk National University, Iksan 54531, South Korea. ^3^The Laboratory of Viromics and Evolution, Korea Zoonosis Research Institute, Jeonbuk National University, 820-120 Hana-ro, Iksan-si, Jeollabuk-do, 54531, Republic of Korea

^4^Department of Biomedical Science, School of Medicine, Kyungpook National University, Daegu, 41944, Republic of Korea

^5^Department of Molecular Medicine, Cell and Matrix Research Institute (CMRI), School of Medicine, Kyungpook National University, Daegu, 41944, Republic of Korea

^6^Department of Physics and Chemistry, Daegu Gyeongbuk Institute of Science and Technology (DGIST), Daegu, 42988, Republic of Korea

^7^New Biology Research Center, Daegu Gyeongbuk Institute of Science and Technology (DGIST), Daegu, 42988, Republic of Korea

^†^These authors contributed equally to this work.

**Correspondence**

Kyungmoo Yea, Department of New Biology and New Biology Research Center, Daegu Gyeongbuk Institute of Science and Technology (DGIST), Daegu, 42988, Republic of Korea

E-mail: [ykm31@dgist.ac.kr](mailto:ykm31@dgist.ac.kr)

Moon-Chang Baek, Department of Molecular Medicine, Cell and Matrix Research Institute (CMRI), School of Medicine, Kyungpook National University, Daegu, 41944, Republic of Korea

E-mail: [mcbaek@knu.ac.kr](mailto:mcbaek@knu.ac.kr)

Kyungmoo Yea and Moon-Chang Baek contributed equally to this work

**Supporting Figures**

**
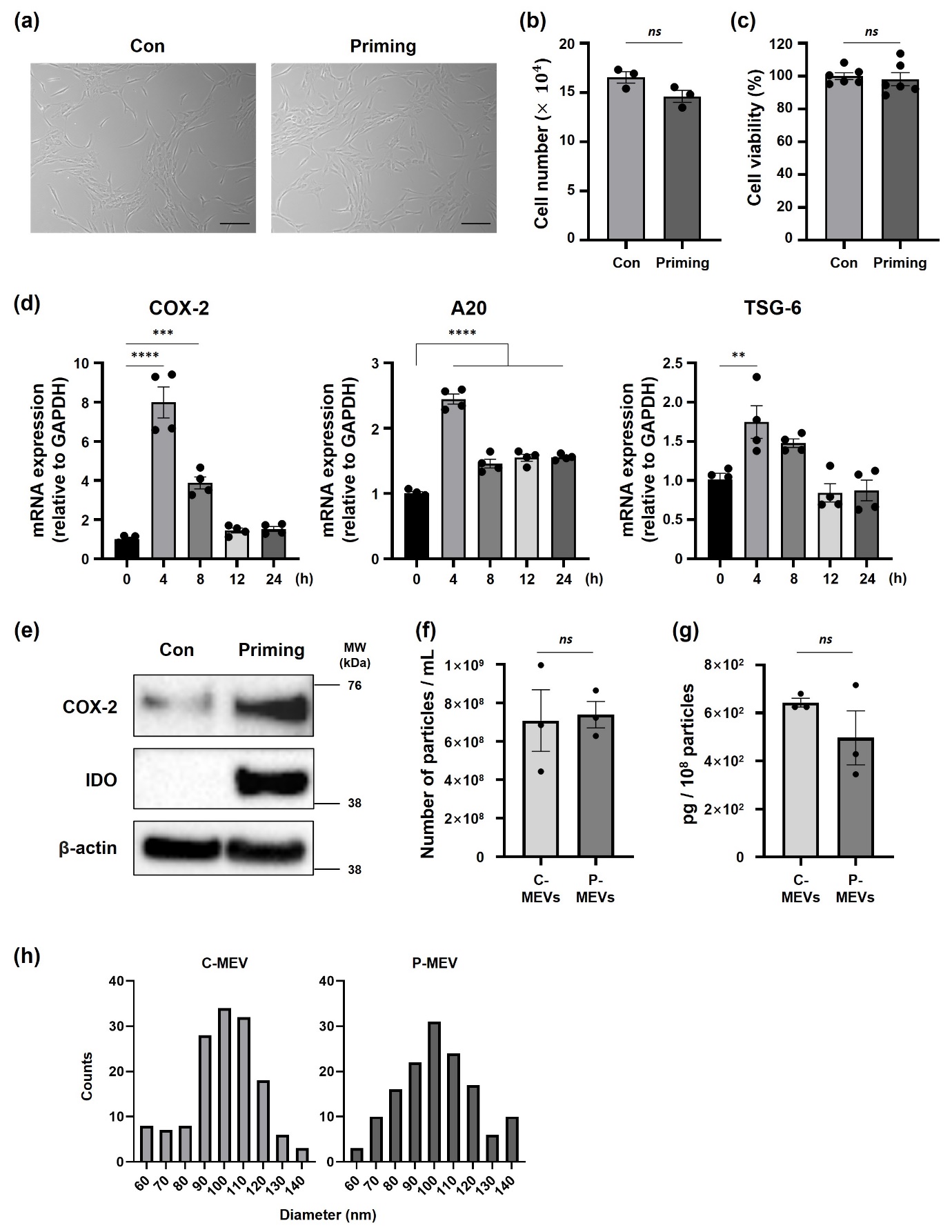
**

**Supplementary figure 1.** Characterization and immunomodulatory profiles of control and primed hADMSCs.

(a) Representative phase contrast images of control (Con) and primed (Priming) hADMSCs after 24 h incubation with IFN-γ (10 ng/mL) and TNF-α (15 ng/mL). Black bars represent 50 μm. (b) Cell proliferation determined by automated cell counting after 24 h of priming. *n =* 3. (c) Cell viability determined by MTS assay after 24 h of priming. *n =* 6. (d) mRNA expression levels of *COX-2*, *A20*, and *TSG-6* over a given period of priming. *n =* 4. (e) Protein expression analysis of COX2, IDO, and β-actin after 24 h of priming (Full-length blots are presented in Supplementary Figure S8b). (f) The number of particles per curtured volume (mL). *n =* 3. (g) A bar graph measuring the protein content of C-MEVs and P-MEVs, presented as picograms (pg) of protein per 10^8^ particles. *n =* 3. (h) Histograms displaying the size distribution of C-MEVs and P-MEVs. *n =* 150. Data are presented as the mean ± standard error of the mean (SEM), analyzed by unpaired two-tailed Student's *t*-test for (b), (c), (f) and (g), and analyzed with a one-way ANOVA, followed by the Holm–Sidak multiple comparison test for (d). Statistical differences in post hoc tests are indicated as *ns* = not significant, ***p* < 0.01, ****p* < 0.001, and *****p* < 0.0001.


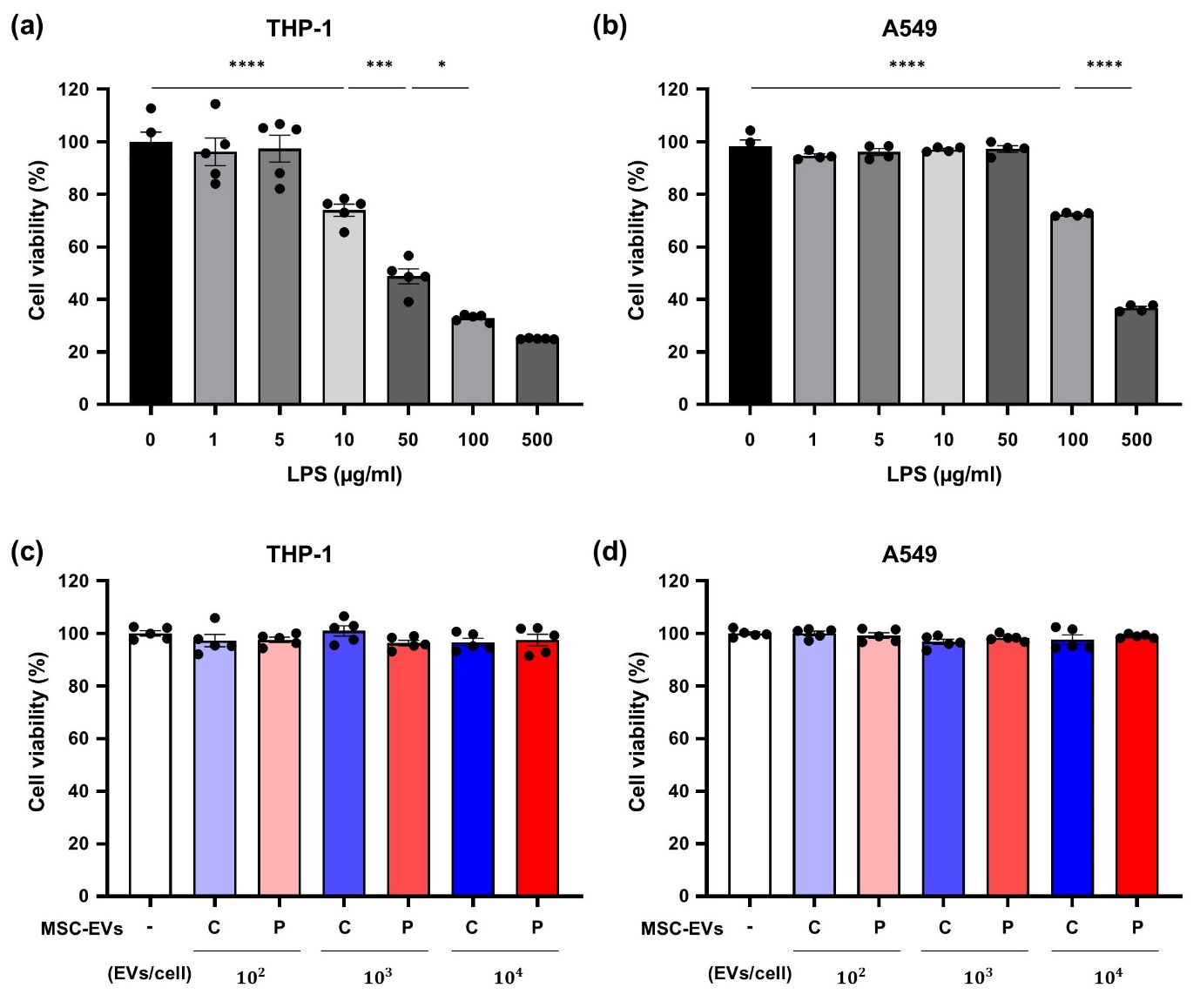


**Supplementary figure 2.** Effects of hADMSC-derived EVs and LPS on cell viability.

(a-d) Cell viability of THP-1 and A549 cells was evaluated by MTS assay. (a) THP-1 cells were treated with the indicated concentrations of LPS for 24 h. *n =* 5. (b) A549 cells were treated with the indicated concentrations of LPS for 24 h. *n =* 4. (c) THP-1 cells were treated with the indicated doses of C-MEVs or P-MEVs for 24 h. *n =* 5. (d) A549 cells were treated with the indicated doses of C-MEVs or P-MEVs for 24 h. *n =* 5. C, control; P, primed. Data are presented as the mean ± SEM, analyzed by one-way ANOVA, followed by the Holm–Sidak multiple comparison test. Statistical differences in post hoc tests are indicated as **p* < 0.05, ****p* < 0.001, and *****p* < 0.0001.


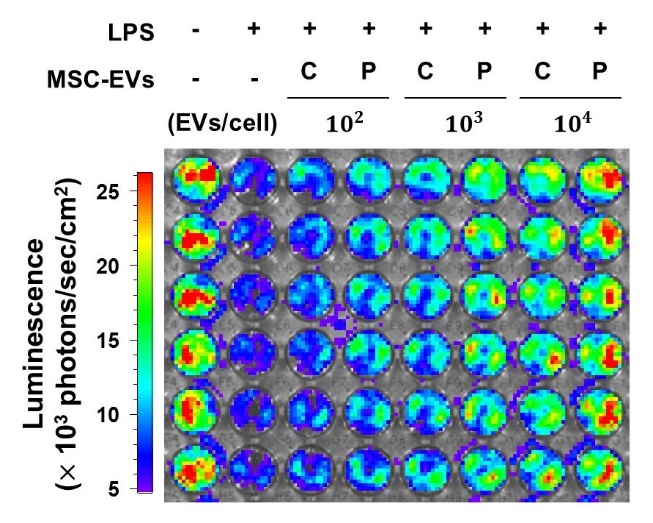


**Supplementary figure 3.** Evaluation of cell viability in lung epithelial cells.

A549 cells were pretreated with MSC-EVs (C-MEVs or P-MEVs) or PBS for 2 h, followed by exposure to LPS (500 μg/mL) for 24 h. Bioluminescence images of A549 cells were captured using an in vivo imaging system (IVIS). C, control; P, primed.


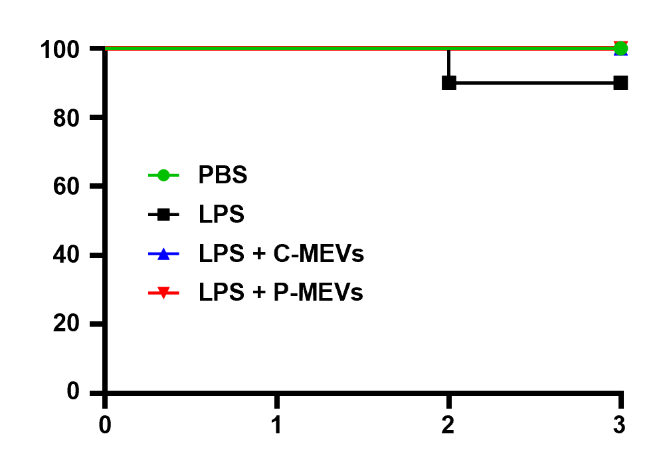


**Supplementary figure 4.**

Summary of survival outcomes during the 72-hour post-treatment period. One mouse (10%) in the LPS group died on day 2. All mice in the C-MEV and P-MEV groups survived throughout the observation period (*n =* 10 per group).


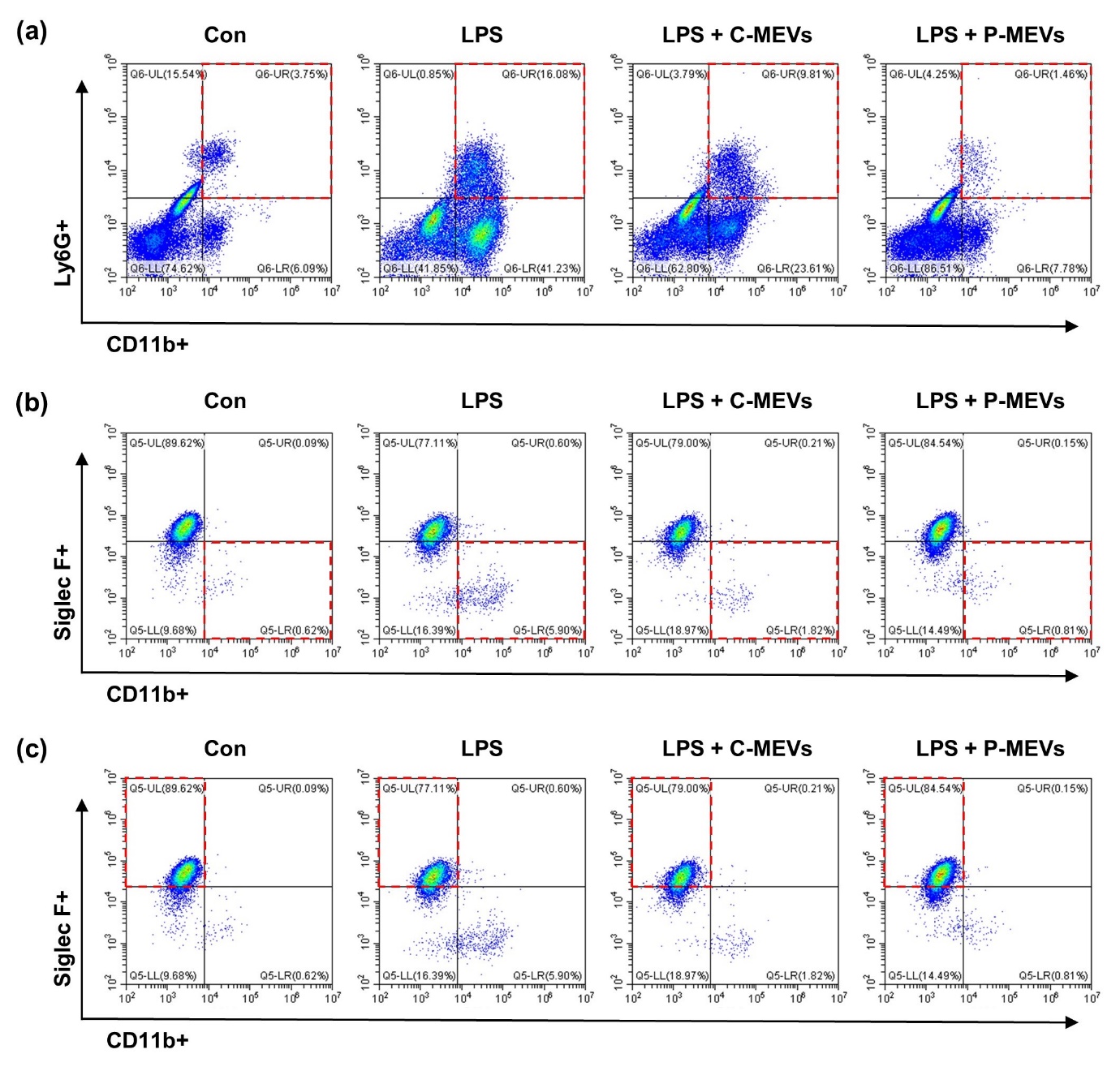


**Supplementary figure 5.** Flow cytometric analysis of immune cell accumulation in lung tissue.

C57BL/6 mice were challenged with LPS (5 mg/kg, intravenously), and then received daily injections of PBS or MSC-EVs (6 $\times{10}^{9}$ particles, intravenously). Flow cytometric analysis of (a) neutrophils (CD11b+ Ly6G+), (b) monocyte-derived macrophages (Siglec F- CD11b+ on CD11c+ F4/80+), and (c) alveolar macrophages (Siglec F+ CD11b- on CD11c+ F4/80+) accumulation in lung tissue at 72 h after LPS injection. C-MEVs, control MSC-EVs; P-MEVs, primed MSC-EVs.


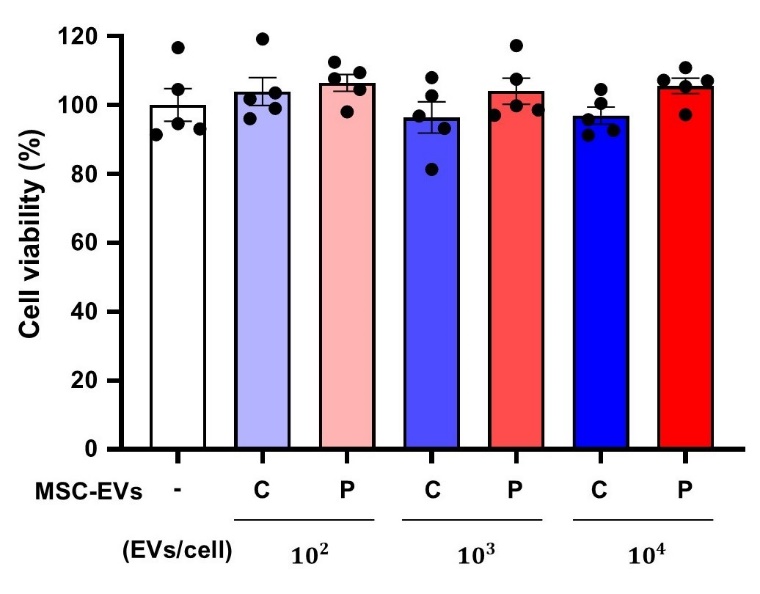


**Supplementary figure 6.** Effects of hADMSC-derived EVs on Vero E6 cell viability.

Cell viability of Vero E6 cells was assessed by the MTS assay. Cells were treated with the indicated doses of C-MEVs or P-MEVs for 48 h. *n =* 5. C, control; P, primed. Data are presented as the mean ± SEM.


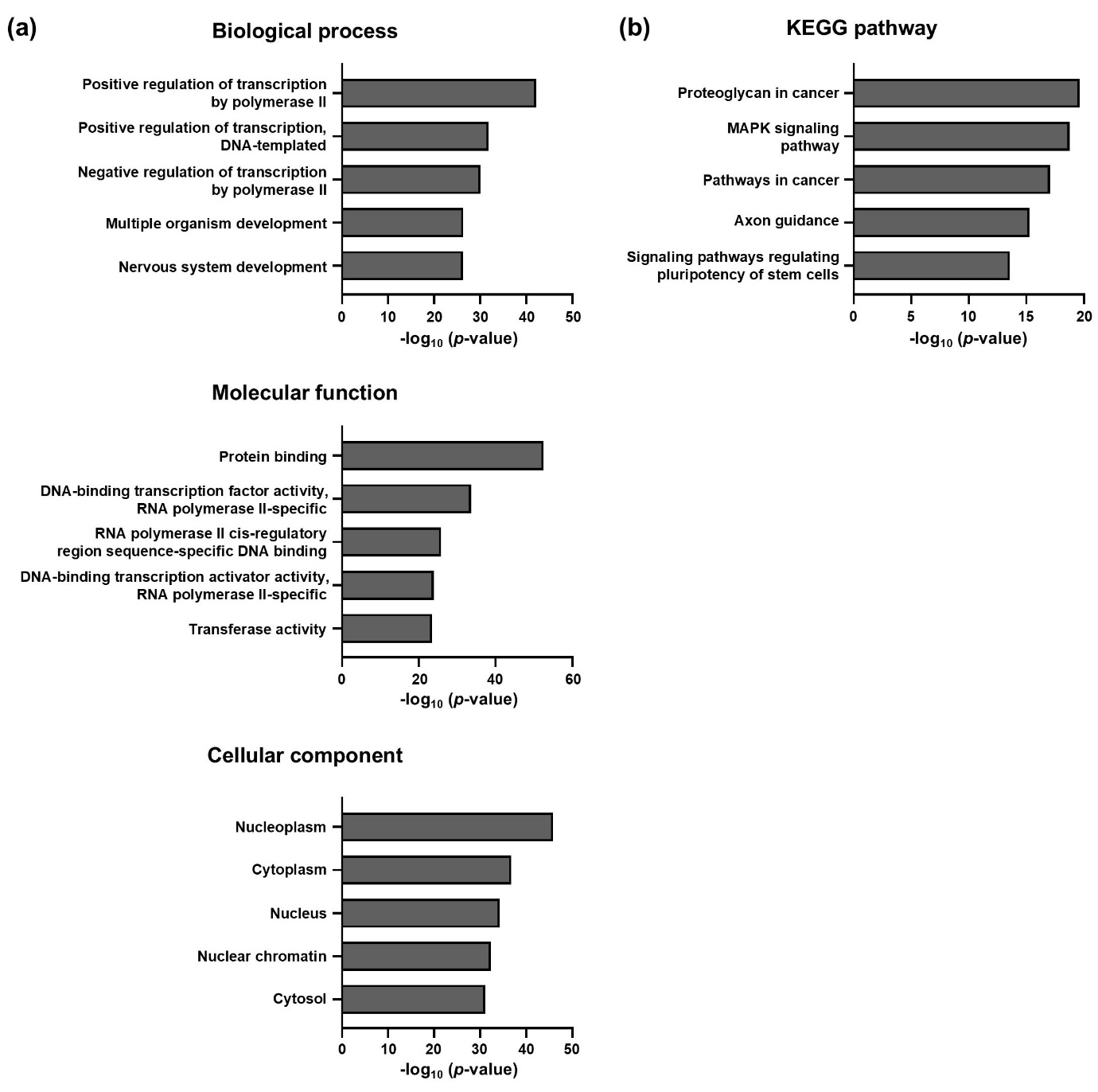


**Supplementary figure 7.** GO and KEGG pathway analysis of the 10 miRNAs highly expressed in P-MEVs.

(A) GO analysis of miRNA target genes. The top five enriched GO terms of the 10 miRNAs highly expressed in P-MEVs. (B) Top five canonical pathways of the 10 miRNAs highly expressed in P-MEVs. P-MEVs, primed MSC-EVs.


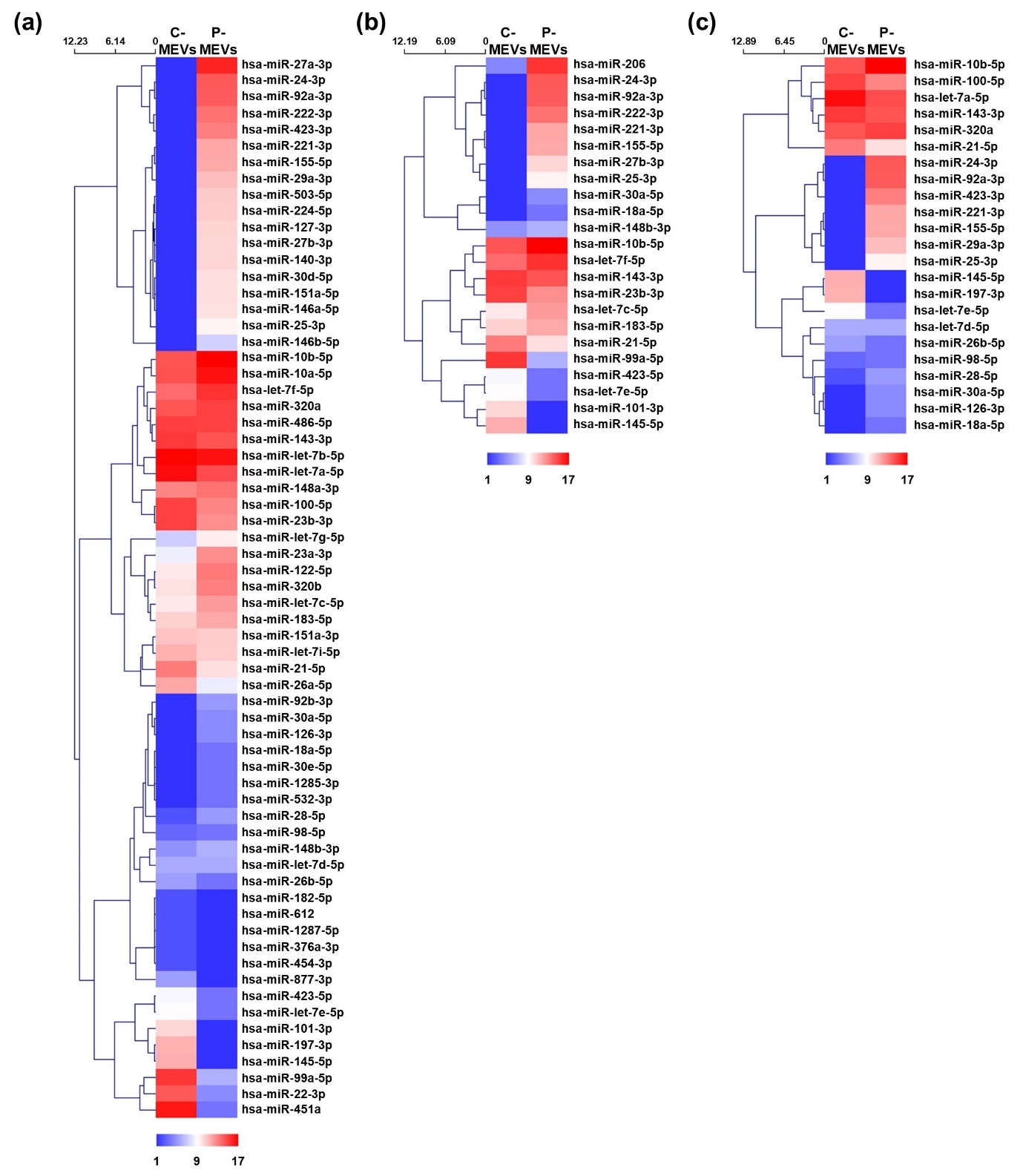


**Supplementary figure 8.** Heatmap analysis of miRNAs in C-MEVs and P-MEVs.

Heatmaps of immune response (A), tissue regeneration (B), and negative regulation of inflammation (C) in C-MEVs and P-MEVs. C-MEVs, control MSC-EVs; P-MEVs, primed MSC-EVs.

**
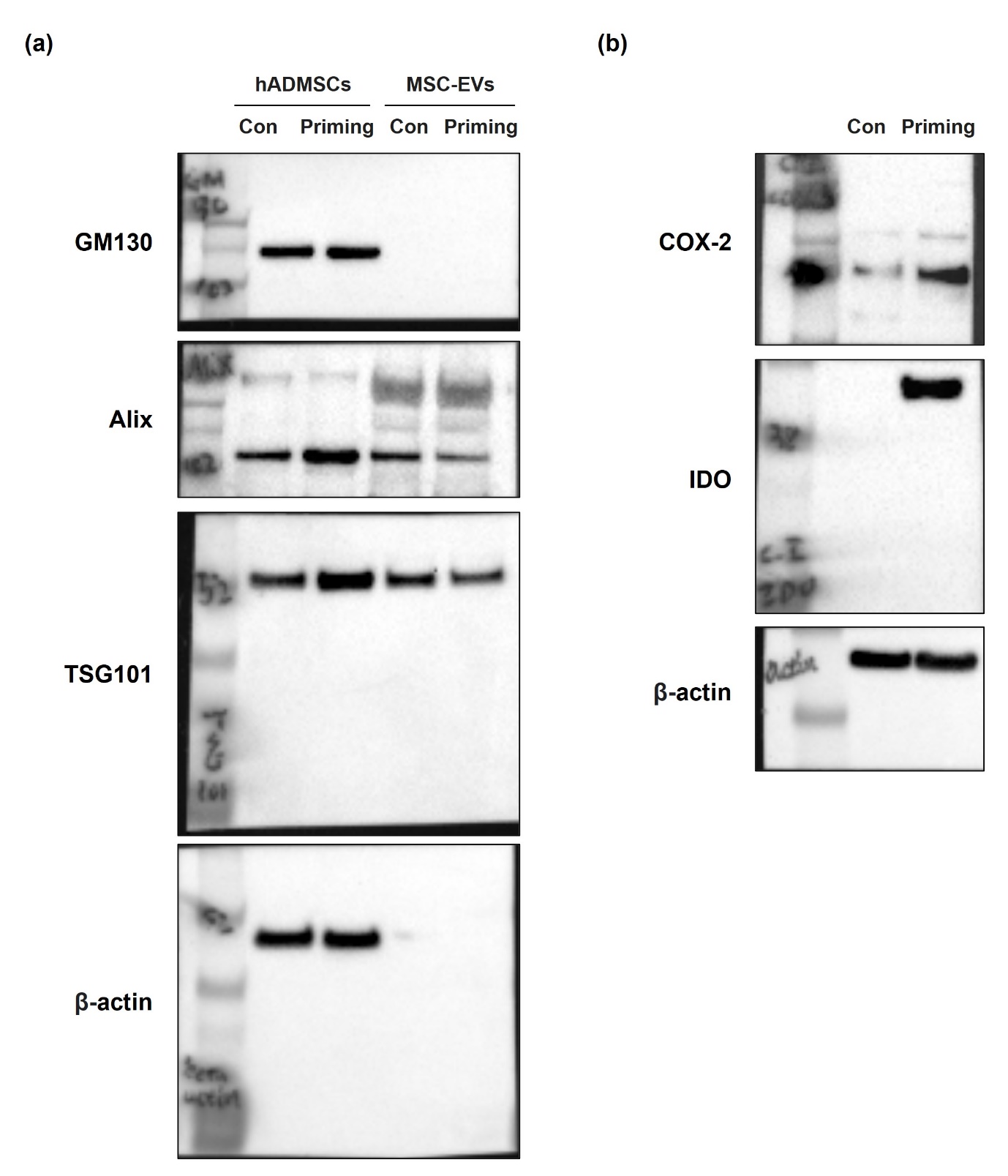
**

**Supplementary figure 9.** Full-length blots.

Full length blots of Figure 1e (A) and Supplementary Figure 1e (B).

**
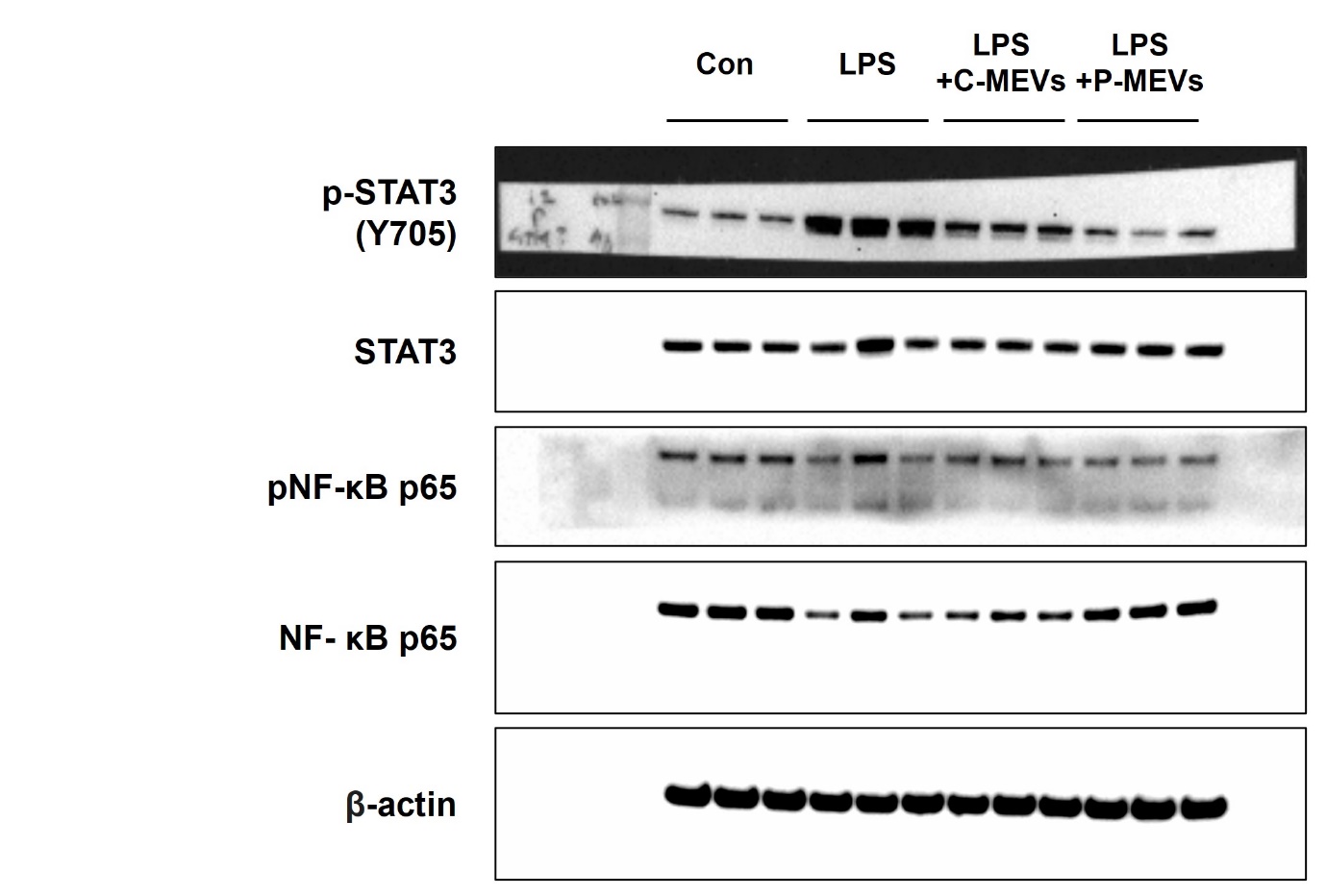
**

**Supplementary figure 10.** Full-length blots.

Full length blots of Figure 4e.

Table S1. List of primers and their sequences for qRT-PCR

| Gene | Species | Forward | Reverse |
| --- | --- | --- | --- |
| TNF*-α* | Human | CTGCTGCACTTTGGAGTGAT | AGATGATCTGACTGCCTGGG |
| IL-1β | Human | GTGGCAATGAGGATGACTTGTTC | TAGTGGTGGTCGGAGATTCGTA |
| IL-6 | Human | AGCCACTCACCTCTTCAGAAC | GCCTCTTTGCTGCTTTCACAC |
| GAPDH | Human | TGCACCACCAACTGCTTAGC | GGCATGGACTGTGGTCATGAG |
| TNF*-α* | Mouse | GGTGCCTATGTCTCAGCCTCTT | GCCATAGAACTGATGAGAGGGAG |
| IL-1β | Mouse | CTTCAGGCAGGCAGTATCACTC | TTGTTGTTCATCTCGGAGCC |
| IL-6 | Mouse | TACCACTTCACAAGTCGGAGGC | CTGCAAGTGCATCATCGTTGTTC |
| GAPDH | Mouse | TCACCACCATGGAGAAGGC | GCTAAGCAGTTGGTGGTGCA |
| TNF*-α* | Monkey | CCTCTCTCTAATCAGCCCTCTG | GAGGACCTGGGAGTAGATGAG |
| IL-1β | Monkey | AGGTCCTGTGCTGAATGTCG | TGGGAGAGGTAAGAGAGGCC |
| IL-6 | Monkey | AAGGAGACATGTAACAGGAG | CTAGGTATACCTCAAACTCC |
| CCL2 | Monkey | CCCCTAGTTTTCCCCTGTTT | CATCCCAGGGATAGAACTGTGG |
| CXCL10 | Monkey | GTGGCATTCAAGGAGTACCTC | GCCTTCGATTCTGGATTCAGACA |
| GAPDH | Monkey | CATGACCACAGTCCACGCCATC | GATGACCTTGCCCACAGCCTTG |
